# Open versus closed suctioning on lung volume and oxygenation in ARDS patients with PEEP 5–10 cmH_2_O: A randomized controlled trial

**DOI:** 10.1371/journal.pone.0351172

**Published:** 2026-09-30

**Authors:** Haotian Gao, Yidie Ying, Jun Yang

**Affiliations:** 1 School of Clinical Medicine, Shandong Second Medical University, Weifang, China; 2 Department of Critical Care Medicine, Third Medical Center of Chinese PLA General Hospital, Beijing, China; 3 Department of Critical Care Medicine, Third Medical Center of Chinese PLA General Hospital, Beijing, China; Scuola Superiore Sant'Anna, ITALY

## Abstract

**Objective:**

To compare the effects of open suctioning (OS) versus closed suctioning (CS) on lung volume and oxygenation in acute respiratory distress syndrome (ARDS) patients receiving mechanical ventilation with positive end-expiratory pressure (PEEP) of 5–10 cmH_2_O.

**Methods:**

This single-center, prospective randomized controlled trial enrolled 60 ARDS patients, who were randomly assigned to OS (n = 30) or CS (n = 30). All patients received lung-protective ventilation with volume-controlled mode. SpO_2_ was recorded before suctioning and at 1, 10, 20, and 30 minutes post-suctioning. Arterial blood gas analysis was performed at 30 minutes to calculate the oxygenation index (PaO_2_/FiO_2_). Changes in end-expiratory lung impedance (ΔEELI) were monitored using electrical impedance tomography (EIT) for the whole lung and four regions of interest (ROI). Secondary outcomes included extubation and survival, analyzed using Kaplan-Meier curves and Cox regression.

**Results:**

The absolute values of global ΔEELI were significantly lower in the CS group than in the OS group at 1 minute (−0.54 ± 0.18 vs. −2.32 ± 1.34, *P* < .001) and 10 minutes (−0.31 ± 0.25 vs. −1.14 ± 0.80, *P* < .001) after suctioning, indicating that CS better preserved lung volume. However, lung volume recovered more rapidly in the OS group, returning to baseline by 20 minutes, whereas recovery in the CS group was not achieved until 30 minutes. Regional analysis showed that the protective effect of CS was primarily observed in non-dependent regions (ROI 1–3), with no significant difference in the gravity-dependent ROI 4 (*P* = .430). SpO_2_ was significantly lower in the OS group than in the CS group at 1 minute post-suctioning (98.07 ± 1.26% vs. 99.03 ± 0.93%, *P* = .007), and returned to baseline by 20 minutes in the OS group versus 10 minutes in the CS group. No significant difference in oxygenation index was observed at 30 minutes (*P* = .329). Secondary outcomes, including extubation and survival, did not differ significantly between the two groups (all *P* > .050). No adverse events were recorded.

**Conclusions:**

In ARDS patients receiving PEEP 5–10 cmH_2_O, CS reduces lung volume loss during suctioning and better preserves early oxygenation, but results in slower post-suctioning lung volume recovery compared with OS. These findings reveal a physiologically important trade-off between the two methods, though their clinical relevance warrants further investigation in larger, outcome-focused trials.

## Introduction

Mechanical ventilation is a cornerstone in the management of acute respiratory distress syndrome (ARDS), and endotracheal suctioning (ES) is critical for maintaining airway patency. The two widely used methods, open suctioning (OS) and closed suctioning (CS), differ in their impact on the breathing circuit and patient physiology—OS requires disconnection from the ventilator, while CS maintains circuit integrity.

ARDS is characterized by high mortality and complex pathophysiology involving alveolar-capillary barrier disruption, alveolar collapse, and V/Q mismatch [1–5]. Current guidelines [6] recommend lung-protective ventilation with low tidal volume, plateau pressure limitation, and appropriate positive end-expiratory pressure (PEEP) to recruit collapsed alveoli and minimize ventilator-induced lung injury (VILI). In this context, any PEEP disruption during suctioning may directly translate into lung volume loss. OS, by requiring ventilator disconnection, inevitably leads to PEEP loss, alveolar collapse, and reduced lung volume [7–9]. In contrast, CS, by maintaining circuit integrity, is theoretically expected to better preserve lung volume and oxygenation [7–10].

However, conflicting findings exist. A study by Heinze et al. [11] demonstrated that regardless of whether CS or OS was used, functional residual capacity decreased after suctioning and remained at reduced levels for up to 20 minutes. Conversely, a study by Corley et al. [8] reported that lung volume recovery was slower following CS compared with OS. These contradictory results suggest that the choice of suctioning technique may be influenced by multiple factors, including the patient’s baseline condition, disease severity, and PEEP level, warranting further investigation.

Notably, electrical impedance tomography (EIT) is a non-invasive, radiation-free imaging technique that enables assessment of changes in end-expiratory lung volume (EELV) by monitoring variations in end-expiratory lung impedance (EELI). This provides a new tool for real-time, bedside quantification of the impact of suctioning on lung volume [12,13].

Current guidelines and studies [7,14] primarily address patients receiving high PEEP (≥10 cmH_2_O), recommending CS in this setting. However, epidemiological data [1] indicate that approximately 50% of mechanically ventilated ARDS patients receive PEEP levels below 10 cmH_2_O—representing a substantial proportion of the ARDS population. For this large patient subgroup, current clinical guidelines offer no clear recommendations, and the limited available evidence remains inconclusive [9,15,16]. Therefore, a dedicated comparison of the safety and efficacy of OS versus CS in this specific population is warranted.

This study focuses on patients with ARDS receiving PEEP levels between 5 and 10 cmH_2_O. By comparing the effects of OS and CS on lung volume and oxygenation, we aim to evaluate the benefits of each suctioning method in this specific patient population and provide evidence to inform clinical practice.

## Methods

### 1 Participants

This study was a single-center, prospective, randomized controlled trial. Patients admitted to the Intensive Care Unit of the Third Medical Center of Chinese Peoples Liberation Army General Hospital between June 1, 2025, and February 28, 2026, were screened for enrollment according to predefined inclusion and exclusion criteria. The study protocol was approved by the institutional ethics committee (No. KY2025−006) and was registered with the Chinese Clinical Trial Registry (registration No. ChiCTR2500105587).

#### 1.1 Inclusion criteria.

(1) Patients receiving mechanical ventilation with a confirmed diagnosis of ARDS according to the Berlin Definition [17];
(2) PEEP level: 5 ≤ PEEP < 10 cmH_2_O;
(3) Age between 18 and 85 years, with written informed consent obtained.

#### 1.2 Exclusion criteria.

(1) PEEP ≥ 10 cmH_2_O or < 5 cmH_2_O;
(2) Age < 18 years or > 85 years;
(3) Pregnancy, presence of an implanted pacemaker, chest trauma or skin lesions precluding placement of the electrode belt, pneumothorax, or terminal illness.

### 2 Study protocol

#### 2.1 Randomisation.

The random allocation sequence was generated by a physician who was not involved in clinical intervention or data collection. Using Microsoft Excel, the physician assigned a sequential number from 1 to 60 to each patient and generated a random number between 0 and 1 for each using the RAND function. Patients were then sorted in ascending order by the random numbers. The first 30 patients were allocated to the open suctioning (OS) group, and the remaining 30 to the closed suctioning (CS) group. The allocation sequence was concealed in sequentially numbered, opaque, sealed envelopes. Patients who met the eligibility criteria were enrolled and assigned according to the envelope sequence.

#### 2.2 Participants and interventions.

Mechanically ventilated patients with ARDS who met the eligibility criteria were enrolled and assigned to receive either open or closed suctioning immediately after enrollment. A total of 60 patients were enrolled and randomly assigned to either the OS group (n = 30) or the CS group (n = 30). All patients received lung-protective ventilation, with volume-controlled mode, tidal volume (Vt) set at 4–8 ml/kg of predicted body weight (PBW), plateau pressure maintained below 30 cmH_2_O, and PEEP titrated by two independent physicians blinded to the study using the ARDSnet low PEEP–FiO_2_ table to determine FiO_2_ and PEEP levels, targeting percutaneous oxygen saturation (SpO_2_) ≥ 88%. Permissive hypercapnia was accepted as part of the ventilation strategy. Adequate sedation and analgesia were administered to all patients, with the Richmond Agitation-Sedation Scale (RASS) maintained between −4 and −5 and the Critical-Care Pain Observation Tool (CPOT) maintained at 0, so as to ensure the absence of any visible spontaneous breathing effort by the patient. No neuromuscular blocking agents were used during the study period. None of the enrolled patients had undergone fiberoptic bronchoscopy or prone positioning prior to enrollment, and none received these interventions during the study period; all patients were maintained in the supine position throughout the suctioning and observation protocol. Except for the suctioning method, all other treatments were identical between the two groups.

#### 2.3 Suctioning procedure.

All suctioning procedures were performed through the patients’ orotracheal tubes by intensive care unit nurses who underwent standardized training, strictly following established suctioning protocols. Prior to suctioning, all patients received 100% oxygen for 60 seconds as pre-oxygenation. The suction pressure was set at −80 to −120 mmHg. The size of both open and closed suction catheters was selected according to the principle that the catheter outer diameter should not exceed one-half of the inner diameter of the artificial airway. Each suctioning pass lasted less than 15 seconds. Normal saline instillation was not routinely performed before suctioning; it was administered only when secretions were thick and viscous and conventional therapy was considered insufficient, in order to facilitate secretion clearance. No recruitment maneuvers were performed after suctioning.

#### 2.4 Outcome measures and data collection.

Demographic characteristics, underlying diseases, Acute Physiology and Chronic Health Evaluation Ⅱ (APACHE Ⅱ) score, Sequential Organ Failure Assessment (SOFA) score, and etiology of ARDS were collected for each patient.

The following parameters were recorded before suctioning (ES-0) and at 1, 10, 20, and 30 minutes after suctioning (ES-1, ES-10, ES-20, ES-30): (1) Oxygenation: percutaneous oxygen saturation (SpO_2_) was recorded, and arterial blood gas analysis was performed at ES-0 and ES-30 to calculate the oxygenation index (PaO_2_/FiO_2_); (2) Lung volume: Changes in end-expiratory lung impedance (ΔEELI) from baseline (ES-0) were continuously monitored using EIT with the Infivision ET1000 device (Hunan Huaruiboshi Medical Equipment Co., Ltd., Xiangtan, China). Global △EELI and regional △EELI for four regions of interest (ROI) were recorded at ES-1, ES-10, ES-20, and ES-30, denoted as △EELI (global) and △EELI (ROI 1–4), respectively. A positive △EELI value indicated an increase in end-expiratory lung volume, whereas a negative value indicated a decrease. In addition, secondary outcomes included successful extubation (no reintubation within 48 hours), mechanical ventilation survival time, and ICU survival time. These data were recorded for all enrolled patients.

Adverse events including desaturation (SpO_2_ < 90%) and arrhythmia, which were systematically recorded during and within 30 minutes after each suctioning procedure.

### 3 Statistical analysis

The modified intention-to-treat (mITT) principle was applied in this study, whereby all randomized patients who received at least one study-specified suctioning procedure were included in the final statistical analysis. Statistical analysis was performed using GraphPad Prism 10.0 software and R 4.5.2. Continuous variables were expressed as mean ± standard deviation or median (interquartile range), and categorical variables were expressed as number (percentage). For baseline comparisons, continuous variables were analyzed using the independent samples t-test or Mann-Whitney U test, and categorical variables were analyzed using the chi-square test or Fisher’s exact test, as appropriate.

A two-way repeated measures analysis of variance was used to analyze the primary outcome measures, with time points (ES-0, ES-1, ES-10, ES-20, ES-30) as the within-subject factor and suctioning method (OS group, CS group) as the between-subject factor. In the presence of significant main effects, simple effects analysis was performed with Bonferroni correction. Comparisons of △EELI values against baseline were performed using one-sample t-tests (test value set to 0). To control for type I error, the significance level was adjusted using the Bonferroni method, with a *P* < .00625 considered statistically significant.

Kaplan–Meier curves with log-rank tests were used to compare mechanical ventilation survival and ICU survival between the two groups. Time on mechanical ventilation was defined as the interval from enrollment to successful extubation or death. Univariate Cox regression was performed to estimate the hazard ratio (*HR*) and 95% confidence interval (*CI*) for the effect of suctioning method on mortality. Extubation outcomes were compared using a Fine-Gray competing-risk model, with death treated as a competing event.

All tests were two-tailed, and a *P* < .050 was considered statistically significant. Bonferroni correction was applied for multiple comparisons.

## Results

### 1 Baseline characteristics

From June 2025 to February 2026, a total of 62 patients with ARDS were admitted to the ICU of our hospital, of whom 60 met the inclusion criteria and were randomly assigned to the OS group (n = 30) or the CS group (n = 30), as shown in Fig 1. Among the enrolled patients, ARDS was caused by pulmonary factors (pulmonary infection) in 56 cases (bacterial infection in 52, viral infection in 3, fungal infection in 1) and by extrapulmonary factors (sepsis) in 4 cases.

**Fig 1:**
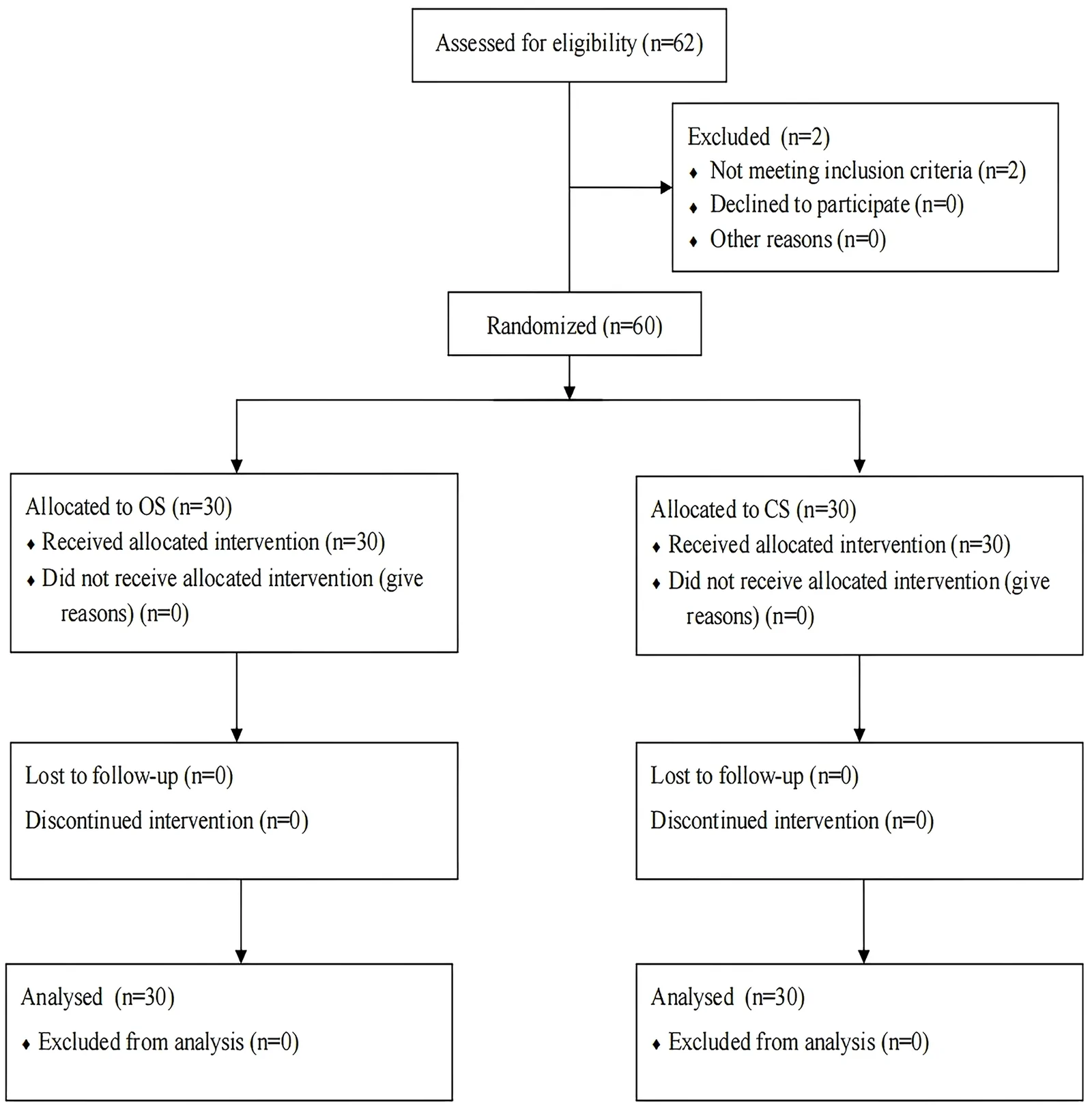
Guidelines Flow Diagram.

Baseline characteristics of the two groups are presented in Table 1. The median (IQR) age of patients in the OS group was 75.00 (65.50, 80.25) years, compared with 69.50 (61.00, 78.25) years in the CS group. The APACHE II mean (SD) scores were 25.00 ± 3.35 and 23.97 ± 5.03, and the SOFA median (IQR) scores were 9.50 (7.00, 13.00) and 9.00 (6.00, 10.50), respectively. No statistically significant differences were observed in baseline characteristics between the two groups.

**Table 1:** Baseline characteristics of the patients.

	Open suctioning (OS)	Closed suctioning (CS)
Age (years)	75.00(65.50, 80.25)	69.50(61.00, 78.25)
Male (n, %)	22(73.33)	20(66.67)
BMI (kg/m^2^)	23.58(19.53, 26.31)	22.55(21.39, 24.83)
Pulmonary etiology (n, %)	27(90.00)	29(96.67)
Medical history		
Hypertension (n, %)	17(56.67)	19(63.33)
Diabetes mellitus (n, %)	9(30.00)	10(33.33)
Coronary heart disease (n, %)	9(30.00)	8(26.67)
Pulmonary disease (n, %)	3(10.00)	4(13.33)
Smoking history (n, %)	11(36.67)	9(30.00)
APACHE Ⅱ score	25.00 ± 3.35	23.97 ± 5.03
SOFA score	9.50(7.00, 13.00)	9.00(6.00, 10.50)
PEEP (cmH_2_O)	6.00(5.00, 7.00)	7.00(5.00, 8.00)
HR (beats/min)	92.20 ± 13.86	88.27 ± 17.68
SBP (mmHg)	128.63 ± 28.22	128.60 ± 23.17
DBP (mmHg)	74.27 ± 16.32	74.43 ± 18.34
CVP (cmH_2_O)	11.23 ± 1.91	11.40 ± 1.98
Pplat (cmH_2_O)	22.93 ± 2.80	23.83 ± 3.32
Pmean (cmH_2_O)	12.75 ± 2.64	13.56 ± 3.21
Cst (ml/cmH_2_O)	22.50 ± 4.58	21.73 ± 3.98
SpO_2_ (%)	99.33 ± 0.92	99.50 ± 0.68
PaO_2_/FiO_2_ (mmHg)	191.38 ± 37.69	196.06 ± 44.73
FiO_2_ (%)	0.55(0.50, 0.60)	0.50(0.50, 0.60)
PaCO_2_ (mmHg)	39.07 ± 6.80	38.40 ± 7.23
Vt (ml)	371.20 ± 45.83	370.83 ± 45.04

Abbreviations: HR, heart rate; SBP, systolic blood pressure; DBP, diastolic blood pressure; CVP, central venous pressure; Pplat, plateau pressure; Pmean, mean airway pressure; Cst, static compliance; Vt, tidal volume

### 2 Primary outcomes

#### 2.1 △EELI (global).

Repeated measures analysis of variance for △EELI (global) revealed significant interaction effect (*F* (1.47, 85.12) = 44.24, *P* < .001), time effect (*F* (1.47, 85.12) = 105.29, *P* < .001), and group effect (*F* (1, 58) = 30.23, *P* < .001).

Further between-group comparisons showed that compared with the CS group, the OS group exhibited a greater decrease in EELI at 1 minute (−2.32 ± 1.34) and 10 minutes (−1.14 ± 0.80) after suctioning (*P* < .001), as shown in Table 2 and Fig 2.

**Table 2:** Intergroup comparison of △EELI (global) post-suctioning.

	ES-1	ES-10	ES-20	ES-30
OS	−2.32 ± 1.34	−1.14 ± 0.80	−0.35 ± 0.66	0.13 ± 0.43
CS	−0.54 ± 0.18	−0.31 ± 0.25	−0.16 ± 0.22	−0.01 ± 0.19
*P*	<.001	<.001	.610	.690
95%*CI*	(−2.44, −1.13)	(−1.24, −0.43)	(−0.52, −0.15)	(−0.11, 0.35)

**Fig 2:**
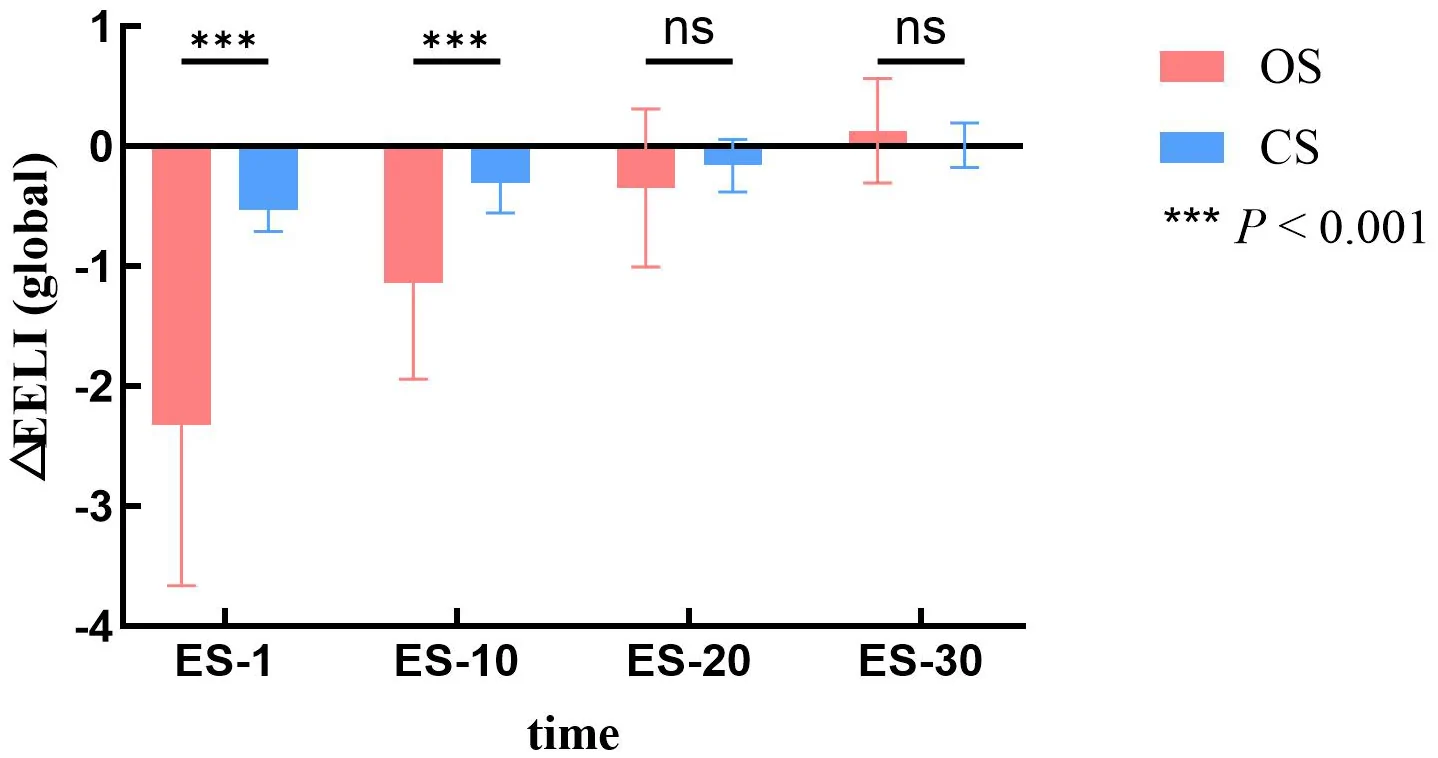
Comparison of △EELI (global) between groups.

Regarding the recovery of EELI after suctioning, the mean △EELI values at the four time points were compared against zero for each group, as values closer to zero indicate a return to baseline. The results demonstrated that EELI recovery was faster in the OS group than in the CS group. In the OS group, EELI returned to baseline by 20 minutes after suctioning (*P* = .007), whereas in the CS group, recovery was not achieved until 30 minutes after suctioning (*P* = .790), as shown in Table 3.

**Table 3:** Post‑suctioning △EELI (global) versus baseline.

Group(time point)	95%*CI*	*t(df)*	*P*
OS(ES-1)	(−2.82, −1.82)	9.48(29.00)	<.000
OS(ES-10)	(−1.44, −0.84)	7.79(29.00)	<.001
OS(ES-20)	(−0.59, −0.10)	2.90(29.00)	.007
OS(ES-30)	(−0.03, 0.29)	1.62(29.00)	.115
CS(ES-1)	(−0.60, −0.47)	16.77(29.00)	<.001
CS(ES-10)	(−0.40, −0.21)	6.69(29.00)	<.001
CS(ES-20)	(−0.24, −0.08)	4.09(29.00)	<.001
CS(ES-30)	(−0.06, 0.08)	0.27(29.00)	.790

Note: The significance threshold was set at *P* < .00625 after Bonferroni correction.

#### 2.2 △EELI (ROI 1).

Analysis of △EELI in ROI 1 revealed significant interaction effect (*F* (1.51, 87.44) = 42.80, *P* < .001), group effect (*F* (1, 58) = 31.85, *P* < .001), and time effect (*F* (1.51, 87.44) = 101.15, *P* < .001).

Compared with the CS group, the OS group exhibited a greater decrease in EELI at 1 minute (−0.83 ± 0.49 vs. −0.17 ± 0.10) and 10 minutes (−0.49 ± 0.38 vs. −0.09 ± 0.07) after suctioning (*P* < .001), as shown in Fig 3A. EELI returned to baseline by 20 minutes after suctioning in both the OS and CS groups (*P* = .007 and *P* = .020, respectively).

**Fig 3:**
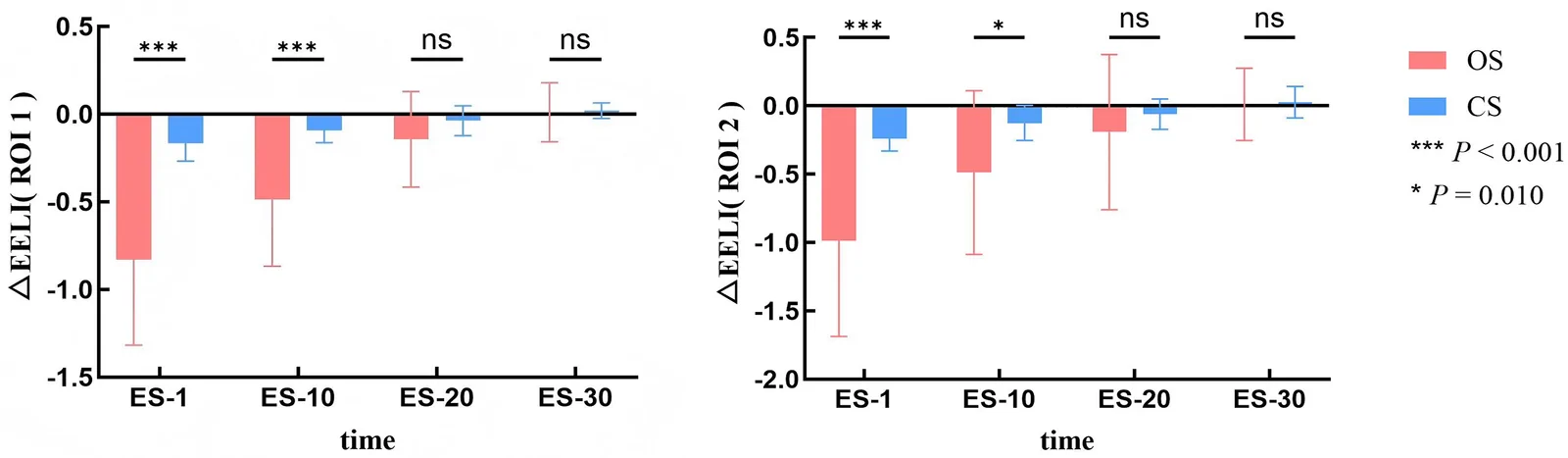
Comparison of △EELI in the non-dependent region between groups. Fig 3A (left) and Fig 3B (right) represent △EELI for ROI 1 and ROI 2, respectively.

#### 2.3 △EELI (ROI 2).

Analysis of △EELI in ROI 2 revealed significant interaction effect (*F* (1.63, 94.54) = 24.84, *P* < .001), group effect (*F* (1, 58) = 12.99, *P* < .001), and time effect (*F* (1.63, 94.54) = 70.29, *P* < .001).

Between-group comparisons showed that compared with the CS group, the OS group showed a greater decrease in EELI at 1 minute (−0.99 ± 0.70 vs. −0.24 ± 0.09, *P* < .001) and 10 minutes (−0.49 ± 0.60 vs. −0.13 ± 0.13, *P* < .010) after suctioning, as shown in Fig 3B. Regarding recovery, EELI returned to baseline by 20 minutes after suctioning in the OS group (*P* = .080), whereas recovery in the CS group was not achieved until 30 minutes (*P* = .220).

#### 2.4 △EELI (ROI 3).

Analysis of changes in EELI in ROI 3, a gravity-dependent region, revealed significant interaction effect (*F* (1.42, 82.61) = 16.18, *P* < .001), group effect (*F* (1, 58) = 7.64, *P* = .008), and time effect (*F* (1.42, 82.61) = 28.75, *P* < .001).

Compared with the CS group, the OS group exhibited a greater decrease in EELI at 1 minute after suctioning (−0.43 ± 0.38 vs. −0.10 ± 0.06, *P* < .001). No statistically significant differences were observed between the two groups at 10, 20, or 30 minutes after suctioning, as shown in Fig 4A. Regarding recovery, EELI returned to baseline by 20 minutes after suctioning in both the OS and CS groups (*P* = .670 and *P* = .080, respectively).

**Fig 4:**
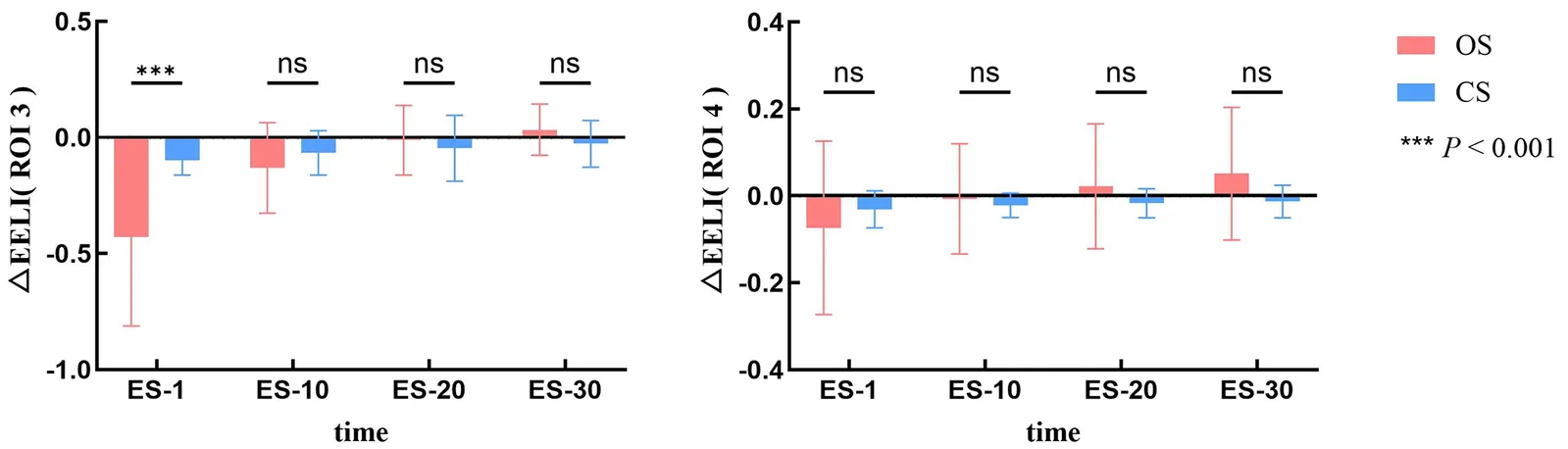
Comparison of △EELI in the gravity-dependent region between groups. Fig 4A (left) and Fig 4B (right) represent △EELI for ROI 3 and ROI 4, respectively.

#### 2.5 △EELI (ROI 4).

Analysis of changes in EELI in ROI 4, a gravity-dependent region, revealed significant interaction effect (*F* (1.92, 111.38) = 4.91, *P* = .010) and time effect (*F* (1.92, 111.38) = 8.80, *P* < .001), whereas the group effect was not significant (*F* (1, 58) = 0.63, *P* = .430).

Between-group comparisons showed no statistically significant differences in the magnitude of EELI decrease at any time point between the two groups, as shown in Fig 4B. Further analysis of changes within each group revealed that EELI in the OS group did not change significantly from baseline at any time point (*P* > .050), whereas EELI in the CS group decreased after suctioning and returned to baseline by 20 minutes (*P* = .009).

#### 2.6 Percutaneous oxygen saturation.

Analysis of SpO_2_ in the two groups revealed a significant interaction effect (*F* (1.95, 113.20) = 15.01, *P* < .001), a non-significant group effect (*F* (1, 58) = 2.22, *P* = .140), and a significant time effect (*F* (1.95, 113.20) = 63.04, *P* < .001).

Further between-group comparisons showed that both groups experienced a significant decrease in oxygenation at 1 minute after suctioning, with SpO_2_ being significantly lower in the OS group (98.07 ± 1.26%) than in the CS group (99.03 ± 0.93%, *P* = .007). SpO_2_ returned to baseline by 10 minutes after suctioning in the CS group (*P* = .170), whereas recovery in the OS group was not achieved until 20 minutes (*P* = .640). The trend of SpO_2_ changes before and after suctioning in the two groups is shown in Fig 5. In the OS group, oxygenation decreased immediately after suctioning and recovered more slowly.

**Fig 5:**
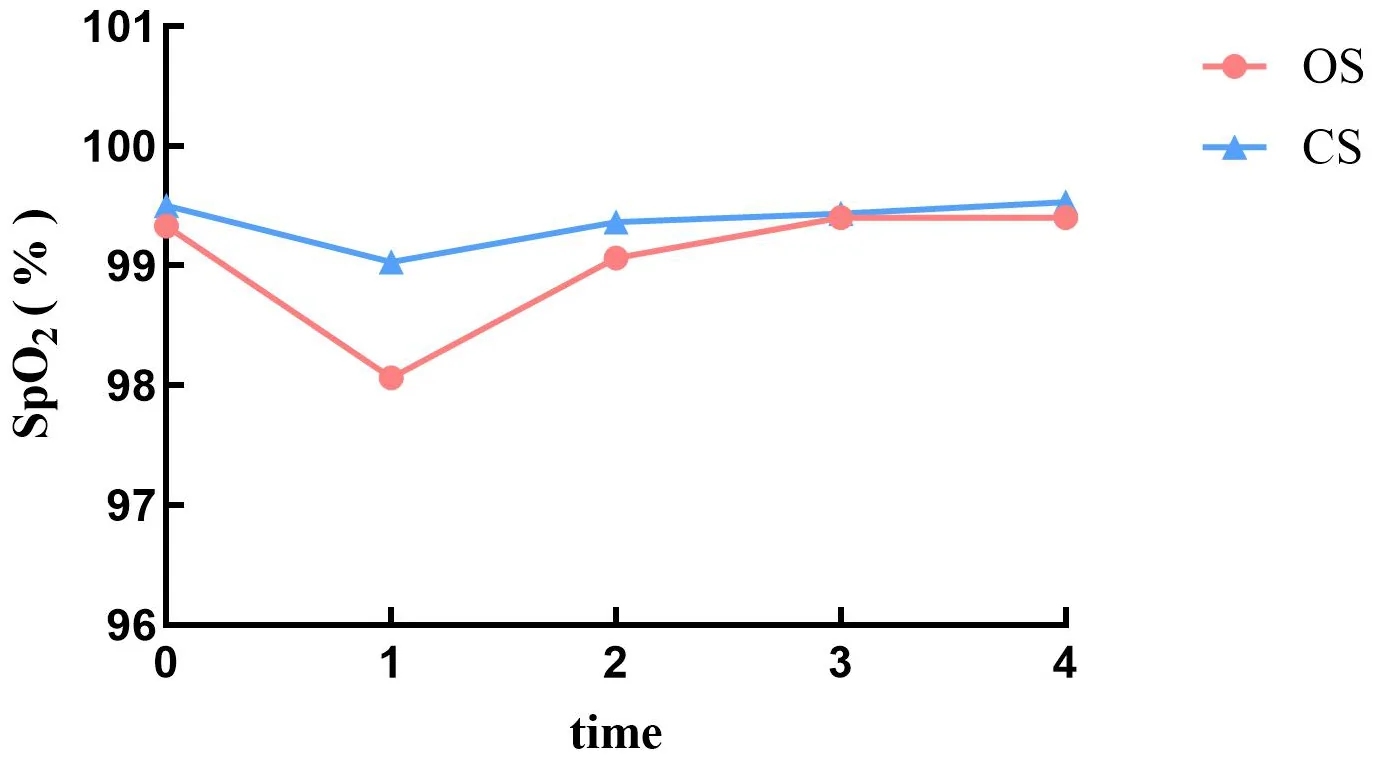
SpO_2_ before and after suctioning. Time points 0, 1, 2, 3, and 4 indicate ES-0, ES-1, ES-10, ES-20, and ES-30, respectively.

#### 2.7 Oxygenation index.

Analysis of the oxygenation index at 30 minutes after suctioning showed values of 188.1 ± 38.46 mmHg in the OS group and 199.6 ± 51.36 mmHg in the CS group, with no significant difference between the two groups (*t* (58) = 0.99, *P* = .329).

### 3 Secondary outcomes

Extubation rates did not differ significantly between the two groups (*P* = .270). Survival analyses were performed using hospital stay and duration of mechanical ventilation as the time-to-event variables. Median hospital survival was 17 days in the OS group and 20 days in the CS group (*P* = .230); univariate Cox regression gave a hazard ratio (*HR*) of 0.67 (95% *CI* (0.35, 1.29), *P* = .228) for CS versus OS. For mechanical ventilation survival, median times were 17 and 20 days, respectively (*P* = .210), with the same *HR* of 0.67 (95%*CI* (0.35, 1.29), *P* = .228). None of these differences reached statistical significance.

### 4 Adverse events

No adverse events were observed during the study period

## Discussion

This randomized controlled trial in ARDS patients receiving PEEP 5–10 cmH_2_O demonstrates that, compared with open suctioning (OS), closed suctioning (CS) better preserves lung volume immediately after suctioning, particularly in non-dependent regions, but lung volume recovery is slower. OS causes a more pronounced early decrease in oxygenation and a slower recovery. These findings reveal a physiologically important trade-off between the two methods that may inform clinical decision-making in this patient population.

### 1 Lung volume: preservation advantage of CS and delayed recovery

For patients with ARDS, appropriate levels of PEEP help maintain the patency of recruitable alveoli and prevent end-expiratory alveolar collapse [18,19]. When PEEP is suddenly discontinued, alveoli that depend on PEEP for patency rapidly collapse, resulting in a significant reduction in lung volume. Concurrently, negative pressure suctioning causes further lung volume loss, exacerbating alveolar collapse. In contrast, CS does not require disconnection from the ventilator during the suctioning procedure and thus maintains continuous PEEP, which theoretically better preserves lung volume [7–9].

EELI is an important parameter derived from EIT, and multiple studies have confirmed a strong linear correlation between EELI and EELV, allowing changes in EELI to represent changes in EELV [20–23]. In the present study, △EELI was used to quantify the impact of the two suctioning methods on lung volume. Consistent with the presumed advantage of CS, the absolute values of △EELI in the CS group were lower than those in the OS group at 1 minute (−0.54 ± 0.18 vs. −2.32 ± 1.34, *P* < .001) and 10 minutes (−0.31 ± 0.25 vs. −1.14 ± 0.80, *P* < .001) after suctioning. Further analysis of △EELI across the four ROI revealed that this lung volume protective effect was primarily observed in ROI 1, 2, and 3, with no significant difference between the two groups in the gravity-dependent ROI 4. This finding holds clinical relevance. In patients with ARDS, increased lung density due to inflammation results in inhomogeneous ventilation distribution along the gravity axis. In the supine position, increased lung density makes the dorsal region (i.e., the gravity-dependent region) more prone to alveolar collapse, and ventilated lung tissue is typically confined to the ventral region [24]. In this study, ROI 4 represented the most dorsal, gravity-dependent region, where alveolar recruitability is poorer and may be less sensitive to changes in lung volume induced by suctioning.

However, this presumed advantage of CS is challenged by the recovery pattern observed after suctioning. Although CS better preserved lung volume immediately after suctioning, the recovery of EELI was slower in the CS group than in the OS group. EELI returned to baseline by 20 minutes after suctioning in the OS group, whereas recovery in the CS group was not achieved until 30 minutes. This finding aligns with the study by Corley et al. [8], who conducted a crossover trial in 20 mechanically ventilated patients after cardiac surgery and similarly found that although CS reduced suction-induced lung volume loss, the recovery of lung volume after suctioning was slower compared with OS. Corley et al. further suggested that this delayed recovery of lung volume following CS may be even more pronounced in patients with ARDS.

The mechanism underlying the delayed recovery of end-expiratory lung volume after CS remains unclear, and the study by Corley et al. did not elucidate it. An in vitro study by El Masry et al. [25] evaluated the response of 11 ventilators to CS and demonstrated that ventilator response to CS varied significantly depending on the ventilation mode and parameter settings. The extent to which ventilator gas delivery is affected by suctioning is closely related to the ventilation mode and tidal volume settings. In the present study, ventilator settings were identical between the two groups, and we did not evaluate the effects of the two suctioning methods on lung volume under other ventilator modes. Given the heterogeneity in ventilator mode settings, suction pressure, and suction catheter size selection across previous studies, further research is needed to elucidate the mechanisms underlying the slower recovery of end-expiratory lung volume following CS. Notably, no adverse events such as desaturation were recorded following suctioning, and secondary clinical outcomes (extubation and survival) did not differ significantly between the two groups. Therefore, the slower recovery of lung volume observed after CS should not be interpreted as evidence of impaired clinical recovery or an increased risk of complications.

In this study, both groups experienced significant lung volume loss after suctioning. Therefore, our findings support the suggestion by Corley et al. [8] that regardless of the suctioning method used, lung recruitment maneuvers may be considered after suctioning to restore lost lung volume and minimize adverse effects such as alveolar collapse and atelectasis. However, lung recruitment maneuvers carry potential risks, including barotrauma, alveolar overdistension, and hemodynamic instability. Therefore, the decision to perform such maneuvers should be tailored to the clinical context, and these risks can be minimized by using strategies such as gradually increasing PEEP in pressure-controlled mode [26].

### 2 Oxygenation: early oxygenation stability with CS

The decrease in percutaneous oxygen saturation is associated with V/Q mismatch resulting from lung volume loss. OS causes loss of PEEP due to ventilator disconnection, leading to alveolar collapse. Some alveoli remain perfused but non-ventilated, resulting in intrapulmonary shunting and reduced oxygenation. CS, by maintaining continuity of the breathing circuit and preserving PEEP levels, better maintains lung volume stability, reduces intrapulmonary shunting, and thereby better preserves oxygenation. A study by Cereda et al. [9] found a significant decrease in percutaneous oxygen saturation after OS. The present study yielded similar results, with SpO_2_ in the OS group decreasing significantly at 1 minute after suctioning (*P* < .001) and returning to baseline by 20 minutes, whereas SpO_2_ in the CS group decreased only at 1 minute and returned to baseline by 10 minutes.

However, it should be noted that these SpO_2_ changes were observed in the context of a standardized pre-oxygenation protocol (100% FiO_2_ for 60 seconds), which may have attenuated the magnitude of desaturation in both groups and contributed to the absence of any clinically significant hypoxemic events during the study period.

Notably, previous studies have reported inconsistent findings. For instance, Lee and Kim [16] observed lower baseline SpO_2_ in CS patients prior to suctioning in a pressure-controlled ventilation setting, a difference that may reflect variations in baseline patient characteristics, ventilation mode, or pre-oxygenation protocols rather than the suctioning method per se. Such discrepancies highlight the influence of contextual factors—including PEEP level, ventilation mode, and pre-oxygenation practice—on the comparative effects of OS and CS, and underscore the need for standardized protocols in future research.

Taken together, these findings suggest that CS may offer an advantage over OS in mitigating early post-suctioning oxygenation decline under the conditions of this study. However, this advantage was transient and did not translate into a sustained difference in oxygenation at 30 minutes. Given that both groups maintained clinically acceptable SpO_2_ levels throughout the observation period, the clinical significance of this early difference remains uncertain and warrants further investigation in larger, outcome-focused trials.

### 3 Study limitations

This study has several limitations. First, the sample size was relatively small, which may have limited the power to detect differences in secondary clinical outcomes such as extubation and survival rates; indeed, we observed no significant differences between the groups in these endpoints (all *P* > .050), consistent with previous reports. Second, due to the nature of the procedure, the suctioning operators and data collectors were not blinded, which may have introduced potential bias. Finally, although our study focused on physiological endpoints, the choice between OS and CS may also have implications for cost and environmental sustainability, which deserve attention in clinical practice. Future studies should incorporate larger, multicenter randomized controlled trials, along with imaging techniques such as chest CT and ultrasound, and include a more comprehensive assessment of clinical outcomes, including ventilator-associated pneumonia, atelectasis, and other pulmonary complications, so as to provide more robust evidence for clinical decision-making.

## Conclusions

In ARDS patients receiving PEEP 5–10 cmH_2_O, CS reduces lung volume loss during suctioning and better preserves early oxygenation, but results in slower post-suctioning lung volume recovery compared with OS. Therefore, the physiological trade-off between the two methods should be considered when selecting a suctioning strategy. There were no significant differences between the two groups in secondary clinical outcomes, including extubation and survival. Whether these physiological differences translate into clinically meaningful outcomes warrants further investigation.

## Supporting information

S1 Table The raw data. (XLSX)

S1 File CONSORT 2025 editable checklist. (DOCX)

S2 File Study Protocol. (DOCX)
