# Supplementary material for: Open versus closed suctioning on lung volume and oxygenation in ARDS patients with PEEP 5–10 cmH2O: A randomized controlled trial
Source: PLoS One. 2026 Sep 30;21(9):e0351172. doi: 10.1371/journal.pone.0351172 (PMC13626340; doi:10.1371/journal.pone.0351172)
Supplement: Supplementary file 2 [file pone.0351172.s003.docx]

**Clinical Study Protocol**

**Study Institution:** Third Medical Center of Chinese PLA General Hospital
**Principal Investigator:** Yang Jun
**Applying Department:** Department of Critical Care Medicine
**Protocol Version:** 2.0
**Date:** 2025-05-23

**1. Study Background**

Mechanical ventilation is a key treatment for patients with acute respiratory distress syndrome (ARDS). Endotracheal suctioning is an essential component of airway management in mechanically ventilated patients, helping to maintain airway patency and prevent ventilator‑associated pneumonia (VAP). The two suctioning methods widely used in clinical practice are open suctioning (OS) and closed suctioning (CS).

ARDS patients are typically managed with lung‑protective ventilation strategies, including low tidal volume, limited plateau pressure, and positive end‑expiratory pressure (PEEP), to keep collapsed alveoli open, prevent alveolar overdistension, reduce ventilator‑induced lung injury, and improve oxygenation. However, OS requires disconnection from the ventilator during the procedure, which theoretically compromises PEEP maintenance. Therefore, some guidelines recommend using CS in patients with PEEP ≥ 10 cmH₂O to maintain alveolar recruitment and reduce lung volume loss.

Nevertheless, epidemiological studies have shown that 82.6% of mechanically ventilated ARDS patients have a PEEP level < 12 cmH₂O, and approximately 50% have a PEEP level < 10 cmH₂O. For these patients, guidelines provide no clear recommendation regarding the choice of suctioning method, and previous studies have reported conflicting results. Further research is therefore needed to determine which suctioning method confers greater benefit in this population.

**2. Study Objectives**

(1) To investigate the effects of open versus closed suctioning on respiratory and circulatory parameters in mechanically ventilated ARDS patients not receiving high PEEP.

(2) To analyze the etiological and clinical characteristics of colonizing versus pathogenic bacteria in patients with lower respiratory tract infections.

**3. Study Methods**

This is a prospective, randomized controlled trial. Mechanically ventilated ARDS patients with PEEP 5–10 cmH₂O will be randomly assigned to receive either open or closed suctioning. Hemodynamic parameters, lung volume, respiratory mechanics, and other variables will be monitored continuously before and after suctioning to compare the immediate effects of the two methods. Bacterial etiological evidence will be collected from patients with lower respiratory tract infections, and their clinical characteristics, treatment regimens, and outcomes will be analyzed.

**4. Trial Protocol**

4.1 Study Population

4.1.1 Inclusion Criteria:

(1) Patients with a confirmed diagnosis of ARDS according to the Berlin Definition who are receiving mechanical ventilation.

(2) PEEP level: 5 ≤ PEEP < 10 cmH₂O.

(3) Age 18–85 years, and written informed consent obtained.

4.1.2 Exclusion Criteria:

(1) PEEP ≥ 10 cmH₂O or PEEP < 5 cmH₂O.

(2) Age < 18 years or > 85 years.

(3) Pregnancy, implanted pacemaker, chest trauma or skin lesions precluding placement of the electrode belt, pneumothorax, or terminal illness.

4.2 Grouping Method

Patients will be randomly assigned using a random number table to either the observation group (closed suctioning) or the control group (open suctioning).

4.3 Baseline Data of Subjects

(1) Age, sex, body mass index (BMI).

(2) Vital signs and laboratory findings: temperature, blood pressure, heart rate, white blood cell count, C‑reactive protein, procalcitonin.

(3) Underlying diseases.

(4) Primary diagnosis.

(5) APACHE II score and SOFA score at enrollment.

4.4 Outcome Measures

4.4.1 Primary outcomes:

(1) Hemodynamics: heart rate (HR), systolic blood pressure (SBP), diastolic blood pressure (DBP), cardiac output (CO), cardiac index (CI), central venous pressure (CVP), pulmonary artery diameter.

(2) Lung volume: tidal volume (Vt), minute ventilation (MV), change in end‑expiratory lung impedance (ΔEELI).

(3) Respiratory mechanics: plateau pressure (Pplat), driving pressure (DP = Pplat – PEEP), mean airway pressure (Pmean), static respiratory system compliance (C = Vt / (Pplat – PEEP)).

(4) Arterial blood gas and oxygenation: pulse oxygen saturation (SpO₂), arterial partial pressure of carbon dioxide (PaCO₂), arterial partial pressure of oxygen (PaO₂), oxygenation index (PaO₂/FiO₂).

4.4.2 Secondary outcomes:

(1) Infection‑related: etiological results (sputum culture / next‑generation sequencing), proportion of multidrug‑resistant organisms.

(2) Treatment: antibiotic use, nebulization, continuous renal replacement therapy (CRRT).

(3) Prognostic outcomes: duration of mechanical ventilation, ICU length of stay, clinical outcome.

**5. Interventions**

(1) After ICU admission and confirmed diagnosis of ARDS, a physician blinded to the study assignment will set the patient’s PEEP level using the PEEP‑FiO₂ table to maintain SpO₂ 88%–95% and PaO₂ 55–80 mmHg. Patients meeting the PEEP inclusion criteria will be randomized according to the random number table.

(2) APACHE II and SOFA scores will be assessed. Baseline data including age, sex, underlying diseases, and primary diagnosis will be recorded. An electrical impedance tomography (EIT) belt with 16 electrodes will be placed around the chest at the 5th or 6th intercostal space to monitor ΔEELI. All patients will be routinely monitored with pulse oximetry for SpO₂, invasive blood pressure monitoring for arterial blood pressure and CVP, and non‑invasive cardiac output monitoring for CO and CI. Pulmonary artery diameter will be measured by ultrasound by the same sonographer each time. Other respiratory mechanics parameters will be obtained from the ventilator. All patients will receive lung‑protective mechanical ventilation in volume‑controlled mode with tidal volume 4–8 ml/kg predicted body weight, maintaining plateau pressure < 30 cmH₂O.

(3) Suctioning will be performed according to standard procedures. Before suctioning, patients will receive 100% oxygen for 60 seconds. Suction pressure will be set at 150 cmH₂O (note: verify clinically; original document stated 150 mmHg, but 150 cmH₂O is the common clinical value). The suction catheter size will be selected such that the catheter outer diameter does not exceed half the inner diameter of the endotracheal tube. Normal saline instillation will not be routinely given before suctioning.

Control group (open suctioning): Sterile technique will be followed. The suction catheter is inserted through the endotracheal tube until resistance is met, then withdrawn 1 cm, negative pressure applied, and the catheter is withdrawn while rotating. Each suctioning episode will not exceed 15 seconds. After suctioning, the ventilator is reconnected.

Observation group (closed suctioning): The suction catheter is inserted through the endotracheal tube while maintaining connection to the ventilator. The suction procedure is the same as for open suctioning.

All suctioning procedures will be performed by trained personnel. Suctioning will be performed on an as‑needed basis.

Lower respiratory tract sputum samples will be collected within 24 hours and sent for analysis within 30 minutes. Sample collection follows standard nursing procedures; no intervention on collection timing is applied. Specimens will be collected according to the *Expert Opinion on Etiological Diagnosis of Adult Respiratory Infectious Diseases* and analyzed by standard microbiological methods plus molecular detection (e.g., resistance genes).

(4) Recording of outcome measures

Primary outcomes will be recorded during the first 72 hours of mechanical ventilation before and after suctioning. All measurements will be performed while the patient is sedated, and ventilator settings will remain unchanged before, during, and after suctioning.

a) Hemodynamic, lung volume, and respiratory mechanics parameters will be recorded before suctioning and at 1, 10, 20, and 30 minutes after suctioning. Arterial blood gas analysis will be performed before suctioning and at 30 minutes after suctioning.

b) All outcome measures will be recorded again at 24, 48, and 72 hours after enrollment, and arterial blood gas will be obtained at those times.

c) The number of suctioning episodes within 24, 48, and 72 hours after enrollment will be recorded.

d) Complications during suctioning (e.g., intratracheal bleeding, arrhythmias) will be recorded.

e) Differences between pre‑ and post‑suctioning values will be calculated and denoted as “Δ”.

Withdrawal criteria: patient extubation, transfer out of the ICU, or clinical need to increase PEEP to ≥10 cmH₂O.

**6. Data Analysis**

Statistical analysis will be performed using SPSS version 26. Continuous data will be expressed as mean ± standard deviation if normally distributed and analyzed by independent samples t‑test; if non‑normally distributed, they will be expressed as median (Q1, Q3) and analyzed by the Mann‑Whitney U test. Categorical data will be expressed as numbers (percentages) and analyzed by the chi‑square test. Cox regression and survival curves will be used for survival analysis. A two‑tailed *P* < 0.05 will be considered statistically significant.
