# Supplementary material for: Open versus closed suctioning on lung volume and oxygenation in ARDS patients with PEEP 5–10 cmH2O: A randomized controlled trial
Source: PLoS One. 2026 Sep 30;21(9):e0351172. doi: 10.1371/journal.pone.0351172 (PMC13626340; doi:10.1371/journal.pone.0351172)
Supplement: Supplementary file 3 [file pone.0351172.s005.docx]

**Manuscript ID:** PONE-D-26-16870
**Title:** Open versus Closed Suctioning on Lung Volume and Oxygenation in ARDS Patients with PEEP 5–10 cmH_2_O: A Randomized Controlled Trial

**Dear Editor and Reviewers,**

We sincerely thank the Editor and the reviewers for their thorough and constructive evaluation of our manuscript. We greatly appreciate the time and effort invested in reviewing our work. We have carefully considered all comments and suggestions, and have revised the manuscript accordingly. Below we provide a point-by-point response to each comment. All changes in the revised manuscript have been highlighted in yellow for easy identification.

**Responses to Academic Editor**

We have carefully addressed all comments raised by both reviewers and we have also formatted the manuscript according to the journal's requirements. We have revised the data availability statement as requested, and the raw data have been added to the supporting information.

**Responses to Reviewer #1**

**Comment 1:** Can the authors follow the CONSORT 2025 reporting guidelines?

**Response:** We thank the reviewer for this suggestion. We have ensured that our manuscript adheres to the CONSORT 2025 reporting guidelines. The flow diagram has been included as Figure 1 (Page 10), and all CONSORT checklist items have been addressed throughout the manuscript. The trial registration number has been provided in the Methods section (Page 5).

**Comment 2:** Statistical analysis section - define population for analysis.

**Response:** We have clarified the analysis population in the Statistical analysis section. We now explicitly state that the modified intention-to-treat (mITT) principle was applied, whereby all randomized patients who received at least one study-specified suctioning procedure were included in the final statistical analysis. (Page 8, lines 169–171)

**Comment 3:** How was missing data handled?

**Response:** We thank the reviewer for this important question. In this study, there were no missing data for the primary outcome measures (ΔEELI and SpO_2_) or the secondary outcomes (extubation and survival), as all measurements were completed according to the protocol. For baseline characteristics, complete data were available for all enrolled patients.

**Comment 4:** Suggest to have a clear outcome section that combining this with data collection, and in the outcomes make clear which is primary and secondary outcomes.

**Response:** We have reorganized the “Outcome Measures and Data Collection” section to clearly distinguish primary and secondary outcomes (Page 8, lines 163–165), and the results are reported separately in the Results section (Page 11–15).

**Comment 5:** Remove p-values from baseline table - as this is an RCT any difference observed would be due to randomness - not recommended to include comparisons in an individually randomised trial.

**Response:** We agree with the reviewer's point. We have removed p-values from the baseline characteristics table in the revised manuscript (Table 1, Page 10–11).

**Comment 6:** Table 1 - replace "OS" and "CS" with actual terms and further abbreviations in the table - either a footnote or stated in full in the table.

**Response:** We have revised Table 1 to replace "OS" and "CS" with the full terms "Open suctioning (OS)" and "Closed suctioning (CS)" in the table header. All other abbreviations in the table have been defined in a footnote. (Table 1, Page 10–11).

**Comment 7:** Sample size calculation is not reported - justification of chosen sample needs to be stated together with what the estimated clinical difference is.

**Response:** We thank the reviewer for the valuable comment on sample size. The sample size was estimated based on the primary outcome—the change in end‑expiratory lung impedance (ΔEELI) at the end of suctioning. According to Yildirim et al. (2024), the mean ΔEELI values were −4415 ± 2363 in the open suctioning group and −2661 ± 1937 in the closed suctioning group. Assuming a two‑sided α = 0.05, power = 80%, and a 10% dropout rate, the calculation yielded 34 patients per group. Owing to the single‑center design, we ultimately enrolled 30 patients per group; however, no dropout occurred during the study, so the effective sample size (30 per group) was close to the target. We acknowledge that the relatively small sample size may have limited the power to detect differences in secondary outcomes, and we have clearly stated this as a limitation in the Discussion section of the revised manuscript (page 19–20, lines 403–406).

**Comment 8:** If the primary comparisons is OS vs CS - what is the correction for and what was the primary timepoint?

**Response:** Since we performed a total of eight comparisons of ΔEELI against a test value of 0 at the 1-, 10-, 20-, and 30-minute time points post-suctioning in the two groups, we applied Bonferroni correction to control for type I error.

**Comment 9:** Line 185 - reporting of age - state what the numbers in brackets are, i.e range/IQR, similar also for APACHE II. Line 187 suggest the sentence starts like "The APACHE II mean (SD) scores were......." "The mean (??) age of patients in OS......"

**Response:** We have revised the reporting of descriptive statistics in the Results section as suggested. For age and SOFA scores, we now clearly indicate that the numbers in parentheses represent the interquartile range (IQR). For APACHE II scores, we report mean ± SD. The sentences have been rephrased accordingly. (Page 10, lines 201–204)

**Comment 10:** The authors used a repeated measures ANOVA - why not use the mixed effect model?

**Response:** We thank the reviewer for this question. We chose repeated measures ANOVA because our data were complete (no missing values) and the study design was balanced, with equal group sizes and the same set of time points for all participants. Under these conditions, repeated measures ANOVA is appropriate and widely accepted in the literature.

**Comment 11:** To what extent was blinding observed - can this be stated. Also when was the SAP finalised and was analysis blinded?

**Response:** We acknowledge that, due to the nature of the suctioning procedure, blinding of the suctioning operators and data collectors was not feasible, and we have discussed this as a limitation in the revised manuscript. However, the physicians responsible for PEEP titration were blinded to group allocation. The statistical analysis plan was finalized before data analysis commenced, although the analysis itself was not performed in a blinded manner.

**Responses to Reviewer #2**

**Comment 1 (Introduction and study rationale):** The Introduction is somewhat lengthy. After the general background on ARDS, suctioning methods, and EIT, the central rationale for studying patients receiving low PEEP is not sufficiently emphasized. The authors should more clearly explain why suctioning-related lung-volume changes may be particularly relevant at PEEP levels of 5 to <10 cmH_2_O, why this population is clinically important, and why it remains insufficiently studied compared with patients receiving higher PEEP.

**Response:** We thank the reviewer for this important suggestion. We have revised the Introduction to more clearly and concisely emphasize the rationale for focusing on the PEEP 5–10 cmH_2_O population. We have added explicit statements highlighting that approximately 50% of mechanically ventilated ARDS patients receive PEEP below 10 cmH_2_O, that current guidelines offer no clear recommendations for this substantial group, and that the limited available evidence remains inconclusive. (Page 3–4)

**Comment 2 (Sample-size calculation):** The manuscript does not report a sample-size or power calculation. Please specify the primary outcome used for the calculation, the anticipated effect size and variability, alpha level, power, expected drop-out rate, and the planned number of participants per group.

**Response:** We thank the reviewer for the valuable comment on sample size. The sample size was estimated based on the primary outcome—the change in end‑expiratory lung impedance (ΔEELI) at the end of suctioning. According to Yildirim et al. (2024), the mean ΔEELI values were −4415 ± 2363 in the open suctioning group and −2661 ± 1937 in the closed suctioning group. Assuming a two‑sided α = 0.05, power = 80%, and a 10% dropout rate, the calculation yielded 34 patients per group. Owing to the single‑center design, we ultimately enrolled 30 patients per group; however, no dropout occurred during the study, so the effective sample size (30 per group) was close to the target. We acknowledge that the relatively small sample size may have limited the power to detect differences in secondary outcomes, and we have clearly stated this as a limitation in the Discussion section of the revised manuscript (page 19–20, lines 403–406).

**Comment 3 (Timing of enrolment and study procedures):** The authors should report the time from intubation, ARDS diagnosis, and ICU admission to study enrolment and to the suctioning procedure. It is unclear whether randomisation and the study intervention were performed immediately after inclusion or after a period of clinical stabilisation. This information is important because respiratory mechanics and recruitability may vary substantially during the course of ARDS.

**Response:** We thank the reviewer for the comment. In this study, patients were randomized and enrolled immediately after meeting the inclusion and exclusion criteria following endotracheal intubation, and subsequently received the corresponding suctioning intervention; we have revised the manuscript to clarify this (Page 6, lines 122–123). However, we acknowledge that the time from ARDS diagnosis to enrollment was not recorded.

**Comment 4 (Baseline ARDS severity and respiratory status):** Please report ARDS severity according to the Berlin categories at enrolment and at the time of the study procedure. Baseline respiratory variables should be presented more comprehensively, including tidal volume indexed to predicted body weight, respiratory rate, FiO_2_, ventilatory mode, driving pressure, arterial pH, PaCO_2_, and duration of mechanical ventilation.

**Response:** We thank the reviewer for this valuable suggestion. We have expanded Table 1 to include additional baseline respiratory variables, including FiO_2_, PaCO_2_, and tidal volume (Vt). The ventilatory mode (volume-controlled) has been described in the Methods section (Page 6, line 125). ARDS severity is reflected in Table 1 by the oxygenation index (PaO_2_/FiO_2_). We acknowledge that respiratory rate, driving pressure, arterial pH, and other variables were not recorded in this study.

**Comment 5 (Prone positioning and patient position during EIT assessment):** The manuscript should clarify whether patients had been managed in the prone position before enrolment, whether any were studied after a prone-positioning session, and whether all measurements were performed in the supine position. This is particularly relevant for the interpretation of regional EIT findings and the definition of dependent versus non-dependent regions.

**Response:** We appreciate the reviewer's careful attention to this methodological detail. We have now clarified that none of the enrolled patients had undergone prone positioning prior to enrollment, and none received prone positioning during the study period. All measurements were performed with patients in the supine position. This information has been added to the Methods section. (Page 7, lines 135–137)

**Comment 6 (Airway characteristics and secretion burden):** It is not clear whether suctioning was performed through an orotracheal tube or a tracheostomy cannula. Please report the number of patients with pre-existing tracheostomy, tracheomalacia, airway abnormalities, or other relevant airway conditions. The authors should also clarify whether patients had undergone bronchoscopy before inclusion and whether airway patency or secretion burden was assessed before suctioning. If available, please report secretion volume, viscosity, or a standardized secretion score in both groups.

**Response:** In response to the reviewer's comment, we have clarified that all suctioning procedures were performed through the patients' orotracheal tubes. None of the enrolled patients had pre‑existing tracheostomy, tracheomalacia, or other relevant airway abnormalities, and none had undergone fiberoptic bronchoscopy prior to enrollment. (Page 7, lines 134–136; lines 140–141) Regarding secretion assessment, we have added that normal saline instillation was not performed on a routine basis; it was administered only when secretions were thick and viscous and, in the assessment of the attending nurse, conventional therapy was considered insufficient to facilitate secretion clearance. (Page 7, lines 146–149) We acknowledge, however, that a standardized secretion score was not recorded prior to suctioning in this study.

**Comment 7 (Standardisation of the suctioning procedure):** Although the manuscript states that nurses followed standardized protocols, the actual suctioning procedure is insufficiently described. Please provide details regarding indications for suctioning, routine suctioning frequency per nursing shift, suction pressure, catheter size, catheter-to-tube diameter ratio, suction duration, number of passes, use of saline instillation, pre-oxygenation, ventilator disconnection technique in the OS group, and whether recruitment maneuvers were allowed after suctioning.

**Response:** We have substantially expanded the description of the suctioning procedure in the Methods section. We now provide details on: pre-oxygenation (100% FiO_2_ for 60 seconds), suction pressure (−80 to −120 mmHg), catheter size selection (outer diameter not exceeding one-half of the inner diameter of the artificial airway), suction duration (less than 15 seconds per pass), saline instillation (not routinely performed; only for thick secretions), and that no recruitment maneuvers were performed after suctioning. Regarding the ventilator disconnection technique in the OS group, suctioning was performed after disconnecting the patient from the ventilator circuit. (Page 7, lines 139–149)

**Comment 8 (Ventilatory management and rationale for low PEEP):** The authors state that all patients received lung-protective ventilation, but the protocol is not sufficiently described. Please clarify whether permissive hypercapnia was accepted, whether plateau pressure and driving pressure targets were standardized, and whether neuromuscular blockade was used. The rationale for selecting PEEP values between 5 and <10 cmH_2_O should be explained. In particular, please indicate whether PEEP was selected according to a standardized protocol, ARDSNet tables, clinician judgment, compliance, oxygenation, or transpulmonary-pressure measurements.

**Response:** We have expanded the description of ventilatory management. We now clarify that: (1) permissive hypercapnia was accepted as part of the ventilation strategy; (2) plateau pressure was maintained below 30 cmH_2_O; (3) no neuromuscular blocking agents were used; (4) PEEP was titrated by two independent physicians blinded to the study using the ARDSnet low PEEP-FiO_2_ table to determine FiO_2_ and PEEP levels, targeting SpO₂ ≥ 88%. The rationale for selecting PEEP 5–10 cmH_2_O is that this range represents a substantial proportion of ARDS patients for whom current guidelines offer no clear recommendations. (Page 6, lines 125–134; Page 4, lines 81–87)

**Comment 9 (Co-interventions and assessment of lung morphology):** Please describe relevant co-interventions and whether they were balanced between groups, including prone positioning, recruitment manoeuvres, bronchodilator therapy, inhaled pulmonary vasodilators, corticosteroids, and neuromuscular blocking agents. It would also be useful to state whether patients underwent chest CT or routine lung ultrasound and to report, where available, the degree of atelectasis, consolidation, and recruitability.

**Response:** We have clarified that none of the enrolled patients received prone positioning, fiberoptic bronchoscopy, or neuromuscular blocking agents during the study period. All other treatments were identical between the two groups except for the suctioning method. Regarding lung morphology assessment, we did not perform routine chest CT or lung ultrasound as part of this study protocol; this has been acknowledged as a limitation and a direction for future research. (Page 7, lines 133–138; Page 20, lines 410–414)

**Comment 10 (Clinical outcomes and length of stay):** Even if the trial was not powered for clinical outcomes, the authors should report ICU length of stay, hospital length of stay, duration of mechanical ventilation, ventilator-associated pneumonia, reintubation or tracheostomy where applicable, and mortality.

**Response:** We have reported the available secondary clinical outcomes in the "Secondary Outcomes" section, including extubation rates, hospital survival, and mechanical ventilation survival. ventilator-associated pneumonia data were not systematically collected as part of this study protocol; we have noted this as a direction for future research. (Page 15, lines 299–306; Page 20, lines 410–414)

**Comment 11 (Ethics and informed consent):** The manuscript states that written informed consent was obtained, but it does not specify when consent was obtained, from whom it was obtained, or how consent was managed in deeply sedated patients without decision-making capacity. Please clarify whether consent was obtained from the patient, a legal representative, or through a deferred-consent procedure, and ensure that this is consistent with the ethics approval.

**Response:** Written informed consent was obtained from the patient's legal representative (next of kin) for all enrolled patients, as all patients were deeply sedated and lacked decision-making capacity at the time of enrollment. This is consistent with the ethics approval (No. KY2025-006).

**Comment 12 (Discussion):** The Discussion should be substantially reorganized. It should begin with a concise statement of the main findings, followed by a structured interpretation of each finding. The authors should then compare their results with previous studies of OS and CS, particularly studies conducted in patients receiving higher PEEP. The reasons why CS was associated with less immediate lung-volume loss but slower recovery should be discussed more clearly and cautiously. The current wording occasionally suggests clinical benefits or risks that were not directly measured in this study.

**Response:** We have substantially reorganized and revised the Discussion according to the reviewer's suggestion. Specifically: (1) The Discussion now begins with a concise summary of the principal findings. (2) The Discussion has been structured into clear subsections: "Lung volume: preservation advantage of CS and delayed recovery" and "Oxygenation: early oxygenation stability with CS." (3) We have compared our results with those of previous studies, including studies conducted in patients receiving higher PEEP (Yildirim et al.) and the study by Corley et al., acknowledging both consistencies and discrepancies. (4) The mechanism underlying the slower recovery of lung volume after CS has been discussed more cautiously, with explicit recognition that this is a physiological observation rather than an indicator of clinical harm. In addition, we have removed wording that could be interpreted as claiming clinical benefits not directly measured in this study. (Pages 15–20)

**Comment 13 (Environmental and economic considerations):** In a sustainability perspective, it would be valuable to include at least a descriptive comparison of the costs and material waste associated with closed versus open suctioning. Even a brief discussion of device use, disposable materials, and potential waste generation would improve the clinical relevance of the manuscript. Once these issues have been adequately addressed, the conclusions should be revised to reflect the corrected analyses and the physiological, rather than clinical-outcome, nature of the findings. There are some typos in Table 3.

**Response:** We thank the reviewer for the valuable comments. We have added environmental and sustainability considerations to the Discussion and revised the Conclusions to better reflect the physiological nature of our findings. We are also grateful for the correction of an extra minus sign in Table 3, which has now been fixed.

We sincerely hope that the revised manuscript now meets the standards for publication in PLOS One. We are grateful again to the editor and reviewers for their time and efforts.

Yours sincerely,

Jun Yang

Corresponding author

Department of Critical Care Medicine

Third Medical Center of Chinese PLA General Hospital

Email: [yangjun@301hospital.com.cn](mailto:yangjun@301hospital.com.cn)
