# Supplementary material for: Open versus closed suctioning on lung volume and oxygenation in ARDS patients with PEEP 5–10 cmH2O: A randomized controlled trial
Source: PLoS One. 2026 Sep 30;21(9):e0351172. doi: 10.1371/journal.pone.0351172 (PMC13626340; doi:10.1371/journal.pone.0351172)
Supplement: Peer review history

# Peer review: Decision Letter 0

**DOI:** 10.1371/journal.pone.0351172.r001

**Authors:** Vincenzo Lionetti

29 Jun 2026

Dear Dr. Yang,

Thank you for submitting your manuscript to PLOS ONE. After careful consideration, we feel that it has merit but does not fully meet PLOS ONE’s publication criteria as it currently stands. Therefore, we invite you to submit a revised version of the manuscript that addresses the points raised during the review process.

==============================

**ACADEMIC EDITOR:**  All issues raised by expert reviewers are required.

Please submit your revised manuscript by Aug 13 2026 11:59PM. If you will need more time than this to complete your revisions, please reply to this message or contact the journal office at plosone@plos.org. When you're ready to submit your revision, log on to https://www.editorialmanager.com/pone/ and select the 'Submissions Needing Revision' folder to locate your manuscript file.

- A letter that responds to each point raised by the academic editor and reviewer(s). You should upload this letter as a separate file labeled 'Response to Reviewers'.
- A marked-up copy of your manuscript that highlights changes made to the original version. You should upload this as a separate file labeled 'Revised Manuscript with Track Changes'.
- An unmarked version of your revised paper without tracked changes. You should upload this as a separate file labeled 'Manuscript'.

If you would like to make changes to your financial disclosure, please include your updated statement in your cover letter. Guidelines for resubmitting your figure files are available below the reviewer comments at the end of this letter.

If applicable, we recommend that you deposit your laboratory protocols in protocols.io to enhance the reproducibility of your results. Protocols.io assigns your protocol its own identifier (DOI) so that it can be cited independently in the future. For instructions see: https://journals.plos.org/plosone/s/submission-guidelines#loc-laboratory-protocols. Additionally, PLOS ONE offers an option for publishing peer-reviewed Lab Protocol articles, which describe protocols hosted on protocols.io. Read more information on sharing protocols at https://plos.org/protocols?utm_medium=editorial-email&utm_source=authorletters&utm_campaign=protocols.

As the corresponding author, your ORCID iD is verified in the submission system and will appear in the published article. PLOS supports the use of ORCID, and we encourage all coauthors to register for an ORCID iD and use it as well. Please encourage your coauthors to verify their ORCID iD within the submission system before final acceptance, as unverified ORCID iDs will not appear in the published article. *Only*  the individual author can complete the verification step; PLOS staff *cannot*  verify ORCID iDs on behalf of authors.

We look forward to receiving your revised manuscript.

Kind regards,

Vincenzo Lionetti, M.D., PhD

Academic Editor

PLOS One

**Journal Requirements:**

When submitting your revision, we need you to address these additional requirements.

1. Please ensure that your manuscript meets PLOS ONE's style requirements, including those for file naming. The PLOS ONE style templates can be found at

https://journals.plos.org/plosone/s/file?id=wjVg/PLOSOne_formatting_sample_main_body.pdf and

https://journals.plos.org/plosone/s/file?id=ba62/PLOSOne_formatting_sample_title_authors_affiliations.pdf

2. In the online submission form you indicate that your data is not available for proprietary reasons and have provided a contact point for accessing this data. Please note that your current contact point is a co-author on this manuscript. According to our Data Policy, the contact point must not be an author on the manuscript and must be an institutional contact, ideally not an individual. Please revise your data statement to a non-author institutional point of contact, such as a data access or ethics committee, and send this to us via return email. Please also include contact information for the third party organization, and please include the full citation of where the data can be found.

3. Please include captions for your Supporting Information files at the end of your manuscript, and update any in-text citations to match accordingly. Please see our Supporting Information guidelines for more information: http://journals.plos.org/plosone/s/supporting-information.

4. If the reviewer comments include a recommendation to cite specific previously published works, please review and evaluate these publications to determine whether they are relevant and should be cited. There is no requirement to cite these works unless the editor has indicated otherwise.

[Note: HTML markup is below. Please do not edit.]

Reviewers' comments:

Reviewer's Responses to Questions

**Comments to the Author**

1. Is the manuscript technically sound, and do the data support the conclusions?

Reviewer #1: Yes

Reviewer #2: Yes

**********

2. Has the statistical analysis been performed appropriately and rigorously?

Reviewer #1: Yes

Reviewer #2: Yes

**********

3. Have the authors made all data underlying the findings in their manuscript fully available?

The PLOS Data policy

Reviewer #1: Yes

Reviewer #2: Yes

**********

4. Is the manuscript presented in an intelligible fashion and written in standard English?

Reviewer #1: Yes

Reviewer #2: Yes

**********

Reviewer #1: Interesting article looking at effectiveness for two suctionaning methods on lung volvume and oxygenation in patient with ARDS receicing PEEP levels

Some comments:

1) Can the authors follow the CONSORT 2025 reporting guidelines.

2) Statistical analysis section - define population for analysis

3) How was missing data handled?

4) Suggest to have a clear outcome section that combining this with data collection, and in the outcomes make clear which is primary and secondary outcomes.

5)Remove p-values from baseline table - as this is an RCT any difference observed would be due to randomness - not recommended to include comparisons in an individually randomised trial.

6) Table 1 - replace "OS" and "CS" with actual terms and further abbreviations in the table - either a footnote or stated in full in the table.

7) Sample size calculation is not reported - justification of chosen sample needs to be stated together with what the estimated clinical difference is.

8) If the primary comparisons is OS vs CS - what is the correction for and what was the primary timepoint?

9) Line 185 - reporting of age - state what the numbers in brackets are, i.e range /IQR, similar also for APACHEII. Line 187 suggest the sentence starts like "The APACHE II mean(SD) scores were......." "The mean (??) age of patients in OS...... "

10) The authors used a repeated measures ANOVA - why not use the mixed effect model?

11) To what extent was blinding observed - can this be stated. Also when was the SAP finalised and was analysis blinded?

Reviewer #2: Thank you to the Editor for the opportunity to review this manuscript. The topic is clinically important and deserves careful consideration, given the vulnerability of patients with acute respiratory distress syndrome (ARDS). Overall, the authors have designed and conducted an interesting randomized study using bedside electrical impedance tomography. However, several methodological, statistical, and reporting issues should be addressed before the conclusions can be considered fully supported.

Major comments

Introduction and study rationale

The Introduction is somewhat lengthy. After the general background on ARDS, suctioning methods, and EIT, the central rationale for studying patients receiving low PEEP is not sufficiently emphasized. The authors should more clearly explain why suctioning-related lung-volume changes may be particularly relevant at PEEP levels of 5 to <10 cmH2O, why this population is clinically important, and why it remains insufficiently studied compared with patients receiving higher PEEP.

Sample-size calculation

The manuscript does not report a sample-size or power calculation. Please specify the primary outcome used for the calculation, the anticipated effect size and variability, alpha level, power, expected drop-out rate, and the planned number of participants per group.

Timing of enrolment and study procedures

The authors should report the time from intubation, ARDS diagnosis, and ICU admission to study enrolment and to the suctioning procedure. It is unclear whether randomisation and the study intervention were performed immediately after inclusion or after a period of clinical stabilisation. This information is important because respiratory mechanics and recruitability may vary substantially during the course of ARDS.

Baseline ARDS severity and respiratory status

Please report ARDS severity according to the Berlin categories at enrolment and at the time of the study procedure. Baseline respiratory variables should be presented more comprehensively, including tidal volume indexed to predicted body weight, respiratory rate, FiO2, ventilatory mode, driving pressure, arterial pH, PaCO2, and duration of mechanical ventilation.

Prone positioning and patient position during EIT assessment

The manuscript should clarify whether patients had been managed in the prone position before enrolment, whether any were studied after a prone-positioning session, and whether all measurements were performed in the supine position. This is particularly relevant for the interpretation of regional EIT findings and the definition of dependent versus non-dependent regions.

Airway characteristics and secretion burden

It is not clear whether suctioning was performed through an orotracheal tube or a tracheostomy cannula. Please report the number of patients with pre-existing tracheostomy, tracheomalacia, airway abnormalities, or other relevant airway conditions. The authors should also clarify whether patients had undergone bronchoscopy before inclusion and whether airway patency or secretion burden was assessed before suctioning. If available, please report secretion volume, viscosity, or a standardized secretion score in both groups.

Standardisation of the suctioning procedure

Although the manuscript states that nurses followed standardized protocols, the actual suctioning procedure is insufficiently described. Please provide details regarding indications for suctioning, routine suctioning frequency per nursing shift, suction pressure, catheter size, catheter-to-tube diameter ratio, suction duration, number of passes, use of saline instillation, pre-oxygenation, ventilator disconnection technique in the OS group, and whether recruitment manoeuvres were allowed after suctioning.

Ventilatory management and rationale for low PEEP

The authors state that all patients received lung-protective ventilation, but the protocol is not sufficiently described. Please clarify whether permissive hypercapnia was accepted, whether plateau pressure and driving pressure targets were standardized, and whether neuromuscular blockade was used. The rationale for selecting PEEP values between 5 and <10 cmH2O should be explained. In particular, please indicate whether PEEP was selected according to a standardized protocol, ARDSNet tables, clinician judgment, compliance, oxygenation, or transpulmonary-pressure measurements.

Co-interventions and assessment of lung morphology

Please describe relevant co-interventions and whether they were balanced between groups, including prone positioning, recruitment manoeuvres, bronchodilator therapy, inhaled pulmonary vasodilators, corticosteroids, and neuromuscular blocking agents. It would also be useful to state whether patients underwent chest CT or routine lung ultrasound and to report, where available, the degree of atelectasis, consolidation, and recruitability.

Clinical outcomes and length of stay

Even if the trial was not powered for clinical outcomes, the authors should report ICU length of stay, hospital length of stay, duration of mechanical ventilation, ventilator-associated pneumonia, reintubation or tracheostomy where applicable, and mortality. These variables would provide useful clinical context for the physiological findings.

Ethics and informed consent

The manuscript states that written informed consent was obtained, but it does not specify when consent was obtained, from whom it was obtained, or how consent was managed in deeply sedated patients without decision-making capacity. Please clarify whether consent was obtained from the patient, a legal representative, or through a deferred-consent procedure, and ensure that this is consistent with the ethics approval.

Discussion

The Discussion should be substantially reorganized. It should begin with a concise statement of the main findings, followed by a structured interpretation of each finding. The authors should then compare their results with previous studies of OS and CS, particularly studies conducted in patients receiving higher PEEP. The reasons why CS was associated with less immediate lung-volume loss but slower recovery should be discussed more clearly and cautiously. The current wording occasionally suggests clinical benefits or risks that were not directly measured in this study.

Environmental and economic considerations

In a sustainability perspective, it would be valuable to include at least a descriptive comparison of the costs and material waste associated with closed versus open suctioning. Even a brief discussion of device use, disposable materials, and potential waste generation would improve the clinical relevance of the manuscript. Once these issues have been adequately addressed, the conclusions should be revised to reflect the corrected analyses and the physiological, rather than clinical-outcome, nature of the findings. There are some typos in Table 3

**********

what does this mean?). If published, this will include your full peer review and any attached files.

If you choose “no”, your identity will remain anonymous but your review may still be made public.

**Do you want your identity to be public for this peer review?**  For information about this choice, including consent withdrawal, please see our Privacy Policy

Reviewer #1: No

Reviewer #2: **Yes:**  Raffaele Mandarano

**********

[NOTE: If reviewer comments were submitted as an attachment file, they will be attached to this email and accessible via the submission site. Please log into your account, locate the manuscript record, and check for the action link "View Attachments". If this link does not appear, there are no attachment files.]

To ensure your figures meet our technical requirements, please review our figure guidelines: https://journals.plos.org/plosone/s/figures

You may also use PLOS’s free figure tool, NAAS, to help you prepare publication quality figures: https://journals.plos.org/plosone/s/figures#loc-tools-for-figure-preparation.

NAAS will assess whether your figures meet our technical requirements by comparing each figure against our figure specifications.

# Author comment: Author response to Decision Letter 1

**DOI:** 10.1371/journal.pone.0351172.r002

19 Aug 2026

Dear Editor and Reviewers,

We sincerely thank the Editor and the reviewers for their thorough and constructive evaluation of our manuscript. We greatly appreciate the time and effort invested in reviewing our work. We have carefully considered all comments and suggestions, and have revised the manuscript accordingly. Below we provide a point-by-point response to each comment. All changes in the revised manuscript have been highlighted in yellow for easy identification.

Responses to Academic Editor

We have carefully addressed all comments raised by both reviewers and we have also formatted the manuscript according to the journal's requirements. We have revised the data availability statement as requested, and the raw data have been added to the supporting information.

Responses to Reviewer #1

Comment 1: Can the authors follow the CONSORT 2025 reporting guidelines?

Response: We thank the reviewer for this suggestion. We have ensured that our manuscript adheres to the CONSORT 2025 reporting guidelines. The flow diagram has been included as Figure 1 (Page 10), and all CONSORT checklist items have been addressed throughout the manuscript. The trial registration number has been provided in the Methods section (Page 5).

Comment 2: Statistical analysis section - define population for analysis.

Response: We have clarified the analysis population in the Statistical analysis section. We now explicitly state that the modified intention-to-treat (mITT) principle was applied, whereby all randomized patients who received at least one study-specified suctioning procedure were included in the final statistical analysis. (Page 8, lines 169–171)

Comment 3: How was missing data handled?

Response: We thank the reviewer for this important question. In this study, there were no missing data for the primary outcome measures (ΔEELI and SpO2) or the secondary outcomes (extubation and survival), as all measurements were completed according to the protocol. For baseline characteristics, complete data were available for all enrolled patients.

Comment 4: Suggest to have a clear outcome section that combining this with data collection, and in the outcomes make clear which is primary and secondary outcomes.

Response: We have reorganized the “Outcome Measures and Data Collection” section to clearly distinguish primary and secondary outcomes (Page 8, lines 163–165), and the results are reported separately in the Results section (Page 11–15).

Comment 5: Remove p-values from baseline table - as this is an RCT any difference observed would be due to randomness - not recommended to include comparisons in an individually randomised trial.

Response: We agree with the reviewer's point. We have removed p-values from the baseline characteristics table in the revised manuscript (Table 1, Page 10–11).

Comment 6: Table 1 - replace "OS" and "CS" with actual terms and further abbreviations in the table - either a footnote or stated in full in the table.

Response: We have revised Table 1 to replace "OS" and "CS" with the full terms "Open suctioning (OS)" and "Closed suctioning (CS)" in the table header. All other abbreviations in the table have been defined in a footnote. (Table 1, Page 10–11).

Comment 7: Sample size calculation is not reported - justification of chosen sample needs to be stated together with what the estimated clinical difference is.

Response: We thank the reviewer for the valuable comment on sample size. The sample size was estimated based on the primary outcome—the change in end‑expiratory lung impedance (ΔEELI) at the end of suctioning. According to Yildirim et al. (2024), the mean ΔEELI values were −4415 ± 2363 in the open suctioning group and −2661 ± 1937 in the closed suctioning group. Assuming a two‑sided α = 0.05, power = 80%, and a 10% dropout rate, the calculation yielded 34 patients per group. Owing to the single‑center design, we ultimately enrolled 30 patients per group; however, no dropout occurred during the study, so the effective sample size (30 per group) was close to the target. We acknowledge that the relatively small sample size may have limited the power to detect differences in secondary outcomes, and we have clearly stated this as a limitation in the Discussion section of the revised manuscript (page 19–20, lines 403–406).

Comment 8: If the primary comparisons is OS vs CS - what is the correction for and what was the primary timepoint?

Response: Since we performed a total of eight comparisons of ΔEELI against a test value of 0 at the 1-, 10-, 20-, and 30-minute time points post-suctioning in the two groups, we applied Bonferroni correction to control for type I error.

Comment 9: Line 185 - reporting of age - state what the numbers in brackets are, i.e range/IQR, similar also for APACHE II. Line 187 suggest the sentence starts like "The APACHE II mean (SD) scores were......." "The mean (??) age of patients in OS......"

Response: We have revised the reporting of descriptive statistics in the Results section as suggested. For age and SOFA scores, we now clearly indicate that the numbers in parentheses represent the interquartile range (IQR). For APACHE II scores, we report mean ± SD. The sentences have been rephrased accordingly. (Page 10, lines 201–204)

Comment 10: The authors used a repeated measures ANOVA - why not use the mixed effect model?

Response: We thank the reviewer for this question. We chose repeated measures ANOVA because our data were complete (no missing values) and the study design was balanced, with equal group sizes and the same set of time points for all participants. Under these conditions, repeated measures ANOVA is appropriate and widely accepted in the literature.

Comment 11: To what extent was blinding observed - can this be stated. Also when was the SAP finalised and was analysis blinded?

Response: We acknowledge that, due to the nature of the suctioning procedure, blinding of the suctioning operators and data collectors was not feasible, and we have discussed this as a limitation in the revised manuscript. However, the physicians responsible for PEEP titration were blinded to group allocation. The statistical analysis plan was finalized before data analysis commenced, although the analysis itself was not performed in a blinded manner.

Responses to Reviewer #2

Comment 1 (Introduction and study rationale): The Introduction is somewhat lengthy. After the general background on ARDS, suctioning methods, and EIT, the central rationale for studying patients receiving low PEEP is not sufficiently emphasized. The authors should more clearly explain why suctioning-related lung-volume changes may be particularly relevant at PEEP levels of 5 to <10 cmH2O, why this population is clinically important, and why it remains insufficiently studied compared with patients receiving higher PEEP.

Response: We thank the reviewer for this important suggestion. We have revised the Introduction to more clearly and concisely emphasize the rationale for focusing on the PEEP 5–10 cmH2O population. We have added explicit statements highlighting that approximately 50% of mechanically ventilated ARDS patients receive PEEP below 10 cmH2O, that current guidelines offer no clear recommendations for this substantial group, and that the limited available evidence remains inconclusive. (Page 3–4)

Comment 2 (Sample-size calculation): The manuscript does not report a sample-size or power calculation. Please specify the primary outcome used for the calculation, the anticipated effect size and variability, alpha level, power, expected drop-out rate, and the planned number of participants per group.

Response: We thank the reviewer for the valuable comment on sample size. The sample size was estimated based on the primary outcome—the change in end‑expiratory lung impedance (ΔEELI) at the end of suctioning. According to Yildirim et al. (2024), the mean ΔEELI values were −4415 ± 2363 in the open suctioning group and −2661 ± 1937 in the closed suctioning group. Assuming a two‑sided α = 0.05, power = 80%, and a 10% dropout rate, the calculation yielded 34 patients per group. Owing to the single‑center design, we ultimately enrolled 30 patients per group; however, no dropout occurred during the study, so the effective sample size (30 per group) was close to the target. We acknowledge that the relatively small sample size may have limited the power to detect differences in secondary outcomes, and we have clearly stated this as a limitation in the Discussion section of the revised manuscript (page 19–20, lines 403–406).

Comment 3 (Timing of enrolment and study procedures): The authors should report the time from intubation, ARDS diagnosis, and ICU admission to study enrolment and to the suctioning procedure. It is unclear whether randomisation and the study intervention were performed immediately after inclusion or after a period of clinical stabilisation. This information is important because respiratory mechanics and recruitability may vary substantially during the course of ARDS.

Response: We thank the reviewer for the comment. In this study, patients were randomized and enrolled immediately after meeting the inclusion and exclusion criteria following endotracheal intubation, and subsequently received the corresponding suctioning intervention; we have revised the manuscript to clarify this (Page 6, lines 122–123). However, we acknowledge that the time from ARDS diagnosis to enrollment was not recorded.

Comment 4 (Baseline ARDS severity and respiratory status): Please report ARDS severity according to the Berlin categories at enrolment and at the time of the study procedure. Baseline respiratory variables should be presented more comprehensively, including tidal volume indexed to predicted body weight, respiratory rate, FiO2, ventilatory mode, driving pressure, arterial pH, PaCO2, and duration of mechanical ventilation.

Response: We thank the reviewer for this valuable suggestion. We have expanded Table 1 to include additional baseline respiratory variables, including FiO2, PaCO2, and tidal volume (Vt). The ventilatory mode (volume-controlled) has been described in the Methods section (Page 6, line 125). ARDS severity is reflected in Table 1 by the oxygenation index (PaO2/FiO2). We acknowledge that respiratory rate, driving pressure, arterial pH, and other variables were not recorded in this study.

Comment 5 (Prone positioning and patient position during EIT assessment): The manuscript should clarify whether patients had been managed in the prone position before enrolment, whether any were studied after a prone-positioning session, and whether all measurements were performed in the supine position. This is particularly relevant for the interpretation of regional EIT findings and the definition of dependent versus non-dependent regions.

Response: We appreciate the reviewer's careful attention to this methodological detail. We have now clarified that none of the enrolled patients had undergone prone positioning prior to enrollment, and none received prone positioning during the study period. All measurements were performed with patients in the supine position. This information has been added to the Methods section. (Page 7, lines 135–137)

Comment 6 (Airway characteristics and secretion burden): It is not clear whether suctioning was performed through an orotracheal tube or a tracheostomy cannula. Please report the number of patients with pre-existing tracheostomy, tracheomalacia, airway abnormalities, or other relevant airway conditions. The authors should also clarify whether patients had undergone bronchoscopy before inclusion and whether airway patency or secretion burden was assessed before suctioning. If available, please report secretion volume, viscosity, or a standardized secretion score in both groups.

Response: In response to the reviewer's comment, we have clarified that all suctioning procedures were performed through the patients' orotracheal tubes. None of the enrolled patients had preexisting tracheostomy, tracheomalacia, or other relevant airway abnormalities, and none had undergone fiberoptic bronchoscopy prior to enrollment. (Page 7, lines 134–136; lines 140–141) Regarding secretion assessment, we have added that normal saline instillation was not performed on a routine basis; it was administered only when secretions were thick and viscous and, in the assessment of the attending nurse, conventional therapy was considered insufficient to facilitate secretion clearance. (Page 7, lines 146–149) We acknowledge, however, that a standardized secretion score was not recorded prior to suctioning in this study.

Comment 7 (Standardisation of the suctioning procedure): Although the manuscript states that nurses followed standardized protocols, the actual suctioning procedure is insufficiently described. Please provide details regarding indications for suctioning, routine suctioning frequency per nursing shift, suction pressure, catheter size, catheter-to-tube diameter ratio, suction duration, number of passes, use of saline instillation, pre-oxygenation, ventilator disconnection technique in the OS group, and whether recruitment maneuvers were allowed after suctioning.

Response: We have substantially expanded the description of the suctioning procedure in the Methods section. We now provide details on: pre-oxygenation (100% FiO2 for 60 seconds), suction pressure (−80 to −120 mmHg), catheter size selection (outer diameter not exceeding one-half of the inner diameter of the artificial airway), suction duration (less than 15 seconds per pass), saline instillation (not routinely performed; only for thick secretions), and that no recruitment maneuvers were performed after suctioning. Regarding the ventilator disconnection technique in the OS group, suctioning was performed after disconnecting the patient from the ventilator circuit. (Page 7, lines 139–149)

Comment 8 (Ventilatory management and rationale for low PEEP): The authors state that all patients received lung-protective ventilation, but the protocol is not sufficiently described. Please clarify whether permissive hypercapnia was accepted, whether plateau pressure and driving pressure targets were standardized, and whether neuromuscular blockade was used. The rationale for selecting PEEP values between 5 and <10 cmH2O should be explained. In particular, please indicate whether PEEP was selected according to a standardized protocol, ARDSNet tables, clinician judgment, compliance, oxygenation, or transpulmonary-pressure measurements.

Response: We have expanded the description of ventilatory management. We now clarify that: (1) permissive hypercapnia was accepted as part of the ventilation strategy; (2) plateau pressure was maintained below 30 cmH2O; (3) no neuromuscular blocking agents were used; (4) PEEP was titrated by two independent physicians blinded to the study using the ARDSnet low PEEP-FiO2 table to determine FiO2 and PEEP levels, targeting SpO₂ ≥ 88%. The rationale for selecting PEEP 5–10 cmH2O is that this range represents a substantial proportion of ARDS patients for whom current guidelines offer no clear recommendations. (Page 6, lines 125–134; Page 4, lines 81–87)

Comment 9 (Co-interventions and assessment of lung morphology): Please describe relevant co-interventions and whether they were balanced between groups, including prone positioning, recruitment manoeuvres, bronchodilator therapy, inhaled pulmonary vasodilators, corticosteroids, and neuromuscular blocking agents. It would also be useful to state whether patients underwent chest CT or routine lung ultrasound and to report, where available, the degree of atelectasis, consolidation, and recruitability.

Response: We have clarified that none of the enrolled patients received prone positioning, fiberoptic bronchoscopy, or neuromuscular blocking agents during the study period. All other treatments were identical between the two groups except for the suctioning method. Regarding lung morphology assessment, we did not perform routine chest CT or lung ultrasound as part of this study protocol; this has been acknowledged as a limitation and a direction for future research. (Page 7, lines 133–138; Page 20, lines 410–414)

Comment 10 (Clinical outcomes and length of stay): Even if the trial was not powered for clinical outcomes, the authors should report ICU length of stay, hospital length of stay, duration of mechanical ventilation, ventilator-associated pneumonia, reintubation or tracheostomy where applicable, and mortality.

Response: We have reported the available secondary clinical outcomes in the "Secondary Outcomes" section, including extubation rates, hospital survival, and mechanical ventilation survival. ventilator-associated pneumonia data were not systematically collected as part of this study protocol; we have noted this as a direction for future research. (Page 15, lines 299–306; Page 20, lines 410–414)

Comment 11 (Ethics and informed consent): The manuscript states that written informed consent was obtained, but it does not specify when consent was obtained, from whom it was obtained, or how consent was managed in deeply sedated patients without decision-making capacity. Please clarify whether consent was obtained from the patient, a legal representative, or through a deferred-consent procedure, and ensure that this is consistent with the ethics approval.

Response: Written informed consent was obtained from the patient's legal representative (next of kin) for all enrolled patients, as all patients were deeply sedated and lacked decision-making capacity at the time of enrollment. This is consistent with the ethics approval (No. KY2025-006).

Comment 12 (Discussion): The Discussion should be substantially reorganized. It should begin with a concise statement of the main findings, followed by a structured interpretation of each finding. The authors should then compare their results with previous studies of OS and CS, particularly studies conducted in patients receiving higher PEEP. The reasons why CS was associated with less immediate lung-volume loss but slower recovery should be discussed more clearly and cautiously. The current wording occasionally suggests clinical benefits or risks that were not directly measured in this study.

Response: We have substantially reorganized and revised the Discussion according to the reviewer's suggestion. Specifically: (1) The Discussion now begins with a concise summary of the principal findings. (2) The Discussion has been structured into clear subsections: "Lung volume: preservation advantage of CS and delayed recovery" and "Oxygenation: early oxygenation stability with CS." (3) We have compared our results with those of previous studies, including studies conducted in patients receiving higher PEEP (Yildirim et al.) and the study by Corley et al., acknowledging both consistencies and discrepancies. (4) The mechanism underlying the slower recovery of lung volume after CS has been discussed more cautiously, with explicit recognition that this is a physiological observation rather than an indicator of clinical harm. In addition, we have removed wording that could be interpreted as claiming clinical benefits not directly measured in this study. (Pages 15–20)

Comment 13 (Environmental and economic considerations): In a sustainability perspective, it would be valuable to include at least a descriptive comparison of the costs and material waste associated with closed versus open suctioning. Even a brief discussion of device use, disposable materials, and potential waste generation would improve the clinical relevance of the manuscript. Once these issues have been adequately addressed, the conclusions should be revised to reflect the corrected analyses and the physiological, rather than clinical-outcome, nature of the findings. There are some typos in Table 3.

Response: We thank the reviewer for the valuable comments. We have added environmental and sustainability considerations to the Discussion and revised the Conclusions to better reflect the physiological nature of our findings. We are also grateful for the correction of an extra minus sign in Table 3, which has now been fixed.

We sincerely hope that the revised manuscript now meets the standards for publication in PLOS One. We are grateful again to the editor and reviewers for their time and efforts.

Yours sincerely,

Jun Yang

Attachment Submitted filename: Response to Reviewers.docx

# Peer review: Decision Letter 1

**DOI:** 10.1371/journal.pone.0351172.r003

**Authors:** Vincenzo Lionetti

15 Sep 2026

Open versus Closed Suctioning on Lung Volume and Oxygenation in ARDS Patients with PEEP 5–10 cmH2O: A Randomized Controlled Trial

PONE-D-26-16870R1

Dear Dr. Yang,

We’re pleased to inform you that your manuscript has been judged scientifically suitable for publication and will be formally accepted for publication once it meets all outstanding technical requirements.

Within one week, you’ll receive an e-mail detailing the required amendments. When these have been addressed, you’ll receive a formal acceptance letter and your manuscript will be scheduled for publication.

An invoice will be generated when your article is formally accepted. Please note, if your institution has a publishing partnership with PLOS and your article meets the relevant criteria, all or part of your publication costs will be covered. Please make sure your user information is up-to-date by logging into Editorial Manager at Editorial Manager® and clicking the ‘Update My Information' link at the top of the page. For questions related to billing, please contact billing support.

If your institution or institutions have a press office, please notify them about your upcoming paper to help maximize its impact. If they’ll be preparing press materials, please inform our press team as soon as possible -- no later than 48 hours after receiving the formal acceptance. Your manuscript will remain under strict press embargo until 2 pm Eastern Time on the date of publication. For more information, please contact onepress@plos.org.

Kind regards,

Vincenzo Lionetti, M.D., PhD

Academic Editor

PLOS One

Additional Editor Comments (optional):

Reviewers' comments:

Reviewer's Responses to Questions

**Comments to the Author**

Reviewer #1: All comments have been addressed

Reviewer #2: All comments have been addressed

**********

2. Is the manuscript technically sound, and do the data support the conclusions?

Reviewer #1: Yes

Reviewer #2: Yes

**********

3. Has the statistical analysis been performed appropriately and rigorously?

Reviewer #1: Yes

Reviewer #2: Yes

**********

4. Have the authors made all data underlying the findings in their manuscript fully available?

The PLOS Data policy

Reviewer #1: Yes

Reviewer #2: Yes

**********

5. Is the manuscript presented in an intelligible fashion and written in standard English?

Reviewer #1: Yes

Reviewer #2: Yes

**********

Reviewer #1: (No Response)

Reviewer #2: I have no further comments. This is a well conducted research and all comments have been addressed, nevertheless no meaningfully clinical insight emerges from the trial. It could inform researchers anyway

**********

what does this mean?). If published, this will include your full peer review and any attached files.

If you choose “no”, your identity will remain anonymous but your review may still be made public.

**Do you want your identity to be public for this peer review?**  For information about this choice, including consent withdrawal, please see our Privacy Policy

Reviewer #1: No

Reviewer #2: No

**********

# Editor report: Acceptance letter

**DOI:** 10.1371/journal.pone.0351172.r004

**Authors:** Vincenzo Lionetti

PONE-D-26-16870R1

PLOS One

Dear Dr. Yang,

I'm pleased to inform you that your manuscript has been deemed suitable for publication in PLOS One. Congratulations! Your manuscript is now being handed over to our production team.

At this stage, our production department will prepare your paper for publication. This includes ensuring the following:

* All references, tables, and figures are properly cited

* All relevant supporting information is included in the manuscript submission,

* There are no issues that prevent the paper from being properly typeset

You will receive further instructions from the production team, including instructions on how to review your proof when it is ready. Please keep in mind that we are working through a large volume of accepted articles, so please give us a few days to review your paper and let you know the next and final steps.

Lastly, if your institution or institutions have a press office, please let them know about your upcoming paper now to help maximize its impact. If they'll be preparing press materials, please inform our press team within the next 48 hours. Your manuscript will remain under strict press embargo until 2 pm Eastern Time on the date of publication. For more information, please contact onepress@plos.org.

You will receive an invoice from PLOS for your publication fee after your manuscript has reached the completed accept phase. If you receive an email requesting payment before acceptance or for any other service, this may be a phishing scheme. Learn how to identify phishing emails and protect your accounts at https://explore.plos.org/phishing.

If we can help with anything else, please email us at customercare@plos.org.

Thank you for submitting your work to PLOS One and supporting open access.

Kind regards,

PLOS One Editorial Office Staff

on behalf of

Prof. Vincenzo Lionetti

Academic Editor

PLOS One
